# A Precise Temperature-Responsive Bistable Switch Controlling *Yersinia* Virulence

**DOI:** 10.1371/journal.ppat.1006091

**Published:** 2016-12-22

**Authors:** Aaron Mischa Nuss, Franziska Schuster, Louisa Roselius, Johannes Klein, René Bücker, Katharina Herbst, Ann Kathrin Heroven, Fabio Pisano, Christoph Wittmann, Richard Münch, Johannes Müller, Dieter Jahn, Petra Dersch

**Affiliations:** 1 Department of Molecular Infection Biology, Helmholtz Centre for Infection Research, Braunschweig, Germany; 2 Institute of Microbiology, Technical University Braunschweig, Braunschweig, Germany; 3 Institute of Systems Biotechnology, Saarland University, Saarbrücken, Germany; 4 Institute of Mathematics, Technical University Munich, Munich, Germany; 5 Institute of Computational Biology, Helmholtz Centre Munich, Neuherberg, Germany; Stanford University School of Medicine, UNITED STATES

## Abstract

Different biomolecules have been identified in bacterial pathogens that sense changes in temperature and trigger expression of virulence programs upon host entry. However, the dynamics and quantitative outcome of this response in individual cells of a population, and how this influences pathogenicity are unknown. Here, we address these questions using a thermosensing virulence regulator of an intestinal pathogen (RovA of *Yersinia pseudotuberculosis*) as a model. We reveal that this regulator is part of a novel thermoresponsive bistable switch, which leads to high- and low-invasive subpopulations within a narrow temperature range. The temperature range in which bistability is observed is defined by the degradation and synthesis rate of the regulator, and is further adjustable via a nutrient-responsive regulator. The thermoresponsive switch is also characterized by a hysteretic behavior in which activation and deactivation occurred on vastly different time scales. Mathematical modeling accurately mirrored the experimental behavior and predicted that the thermoresponsiveness of this sophisticated bistable switch is mainly determined by the thermo-triggered increase of RovA proteolysis. We further observed RovA ON and OFF subpopulations of *Y*. *pseudotuberculosis* in the Peyer’s patches and caecum of infected mice, and that changes in the RovA ON/OFF cell ratio reduce tissue colonization and overall virulence. This points to a bet-hedging strategy in which the thermoresponsive bistable switch plays a key role in adapting the bacteria to the fluctuating conditions encountered as they pass through the host’s intestinal epithelium and suggests novel strategies for the development of antimicrobial therapies.

## Author Summary

The ability of pathogens to sense temperature changes when they enter their mammalian hosts from the environment is crucial to optimize their fitness and adjust expression of their virulence programs. Until now it has been assumed that all cells within a population participate in the thermo-triggered adaptive response. Here, we show that a small subpopulation of an enteric pathogen does not follow thermo-induced reprogramming when the bacteria pass the intestinal epithelial layer. Observed heterogeneity is promoted by a new type of bistable switch, implicating a highly precise, thermoresponsive control element. Moreover, we demonstrate that this regulatory implement is important for virulence as it prepares the pathogen for sudden, unpredictable fluctuations encountered during host entry and exit.

## Introduction

Temperature is a prominent signal used by pathogens to adjust their virulence and host survival programs during infection. Different biomolecules can act as thermosensors, including DNA, RNA and regulatory proteins. They all detect changes in temperature through thermally induced conformational changes [1–3]. The velocity and reversibility of thermosensors enable rapid adaptation to the temperature shifts encountered when transitioning between different hosts or environments. The precise thermosensation mechanism of several molecular thermometers was uncovered using population level analyses. However, bulk-scale methods are insufficient for characterizing key features of this process, such as sensor dynamics and quantitative outcome in individual cells. Here, we addressed these features by single-cell level analyses using the *Yersinia* regulator protein RovA as an example for a thermosensing molecule that controls virulence [4, 5].

**Fig 1:**
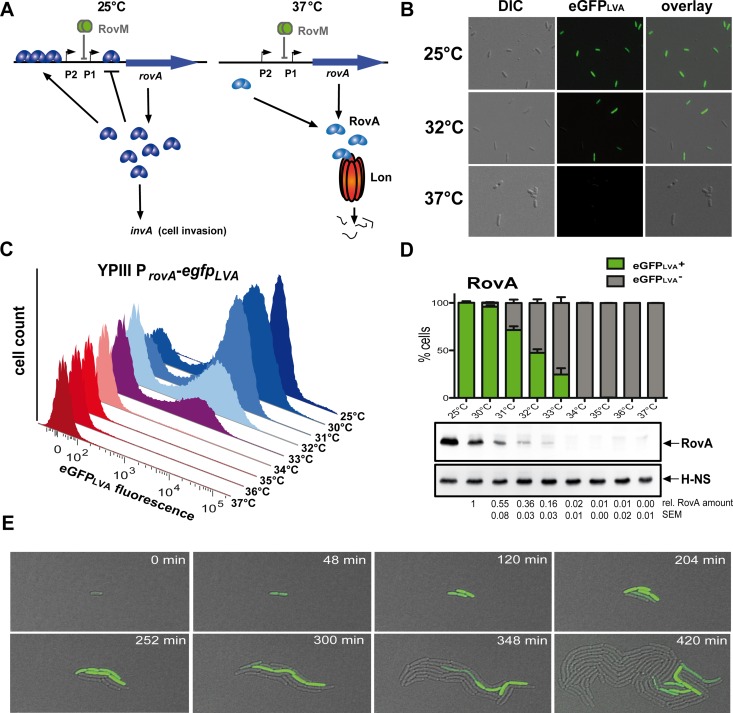
Identification of a temperature-responsive bistable switch. (***A***) The temperature-responsive *Yersinia* virulence regulator RovA is autoregulated through positive and negative feedback loops. At 25°C RovA is active and binds cooperatively to a high-affinity site upstream of P2 and activates *rovA* and *invA* transcription. When the RovA amount has reached a certain threshold, RovA binds to a low affinity site downstream of P1 to prevent uncontrolled *rovA* induction. An upshift to 37°C induces a reversible conformational change in RovA that leads to a strong reduction of its DNA-binding capacity and renders this regulator susceptible to proteolysis by the Lon protease. *rovA* transcription is further regulated by the nutrient-responsive repressor RovM. (***B***) *Y*. *pseudotuberculosis* wild-type carrying a P_*rovA*_-*egfp*_*LVA*_ fusion was grown at different temperatures and analysed by fluorescence microscopy, and (***C***), flow cytometry (one representative replicate; 10^5^ cells). (***D***) The percentage of P_*rovA*_-*egfp*_*LVA*_-expressing wild-type cells quantified by flow cytometry (mean ± SEM; *n* = 3 for each temperature; 10^5^ cells per replicate), eGFP_LVA_-positive cells (ON) are shown in green. The response of P_*rovA*_-*egfp*_*LVA*_ to temperature corresponds to the average RovA level as determined by western blot. Relative RovA amounts were quantified and normalised to the highest temperature for which a homogenous RovA ON population was observed (mean ± SEM; *n* = 3 for each temperature). (***E***), Live cell imaging of *Y*. *pseudotuberculosis* expressing P_*rovA*_-*egfp*_*LVA*_ at 32°C. Time series of individual bacteria starting from the OFF state demonstrates switching to the ON and back to the OFF state; representative overlays of eGFP_LVA_ and bright field images at different time points are shown (also see **S**1 Video).

This approach is important as during transition processes genetically identical populations can generate phenotypic heterogeneity, which supports persistence of pathogens in fluctuating environments (bet-hedging) via fitness improvement of the whole population by cooperativity or division of labor [6–11]. One example is bistability, in which isogenic bacteria exist in two distinct phenotypic states (ON or OFF) driven by divergent gene expression profiles in response to nutrient shifts and stress conditions [6, 10, 12–14]. A binary distribution of phenotypes can be generated by feedback-based circuitry in combination with non-linear responses, e.g. by cooperativity in DNA binding of a regulator, [6, 14], a characteristic also observed for the thermoresponsive virulence regulator RovA. RovA is active and autoregulated at moderate temperatures (20–25°C) and binds cooperatively to a high-affinity site upstream of the distal *rovA* promoter (P2) and activates *rovA* and *invA* transcription. When the RovA amount has reached a certain threshold, RovA binds to a low affinity site downstream of the proximal *rovA* promoter (P1) to prevent uncontrolled *rovA* induction (Fig **1*A***). An upshift to 37°C induces a reversible conformational change in RovA that leads to a strong reduction of its DNA-binding capacity and renders this regulator susceptible to proteolysis by the Lon protease [4, 5, 15] (Fig **1*A***). Since autoregulatory features, which can generate a bistable output of a genetic system, are combined with a thermosensing element [12–14], we hypothesized a novel type of ‘thermo-controllable’ bistable switching device for the control of *Yersinia* virulence.

## Results and Discussion

### A temperature-responsive bistable switching device for control of virulence

**Fig 2:**
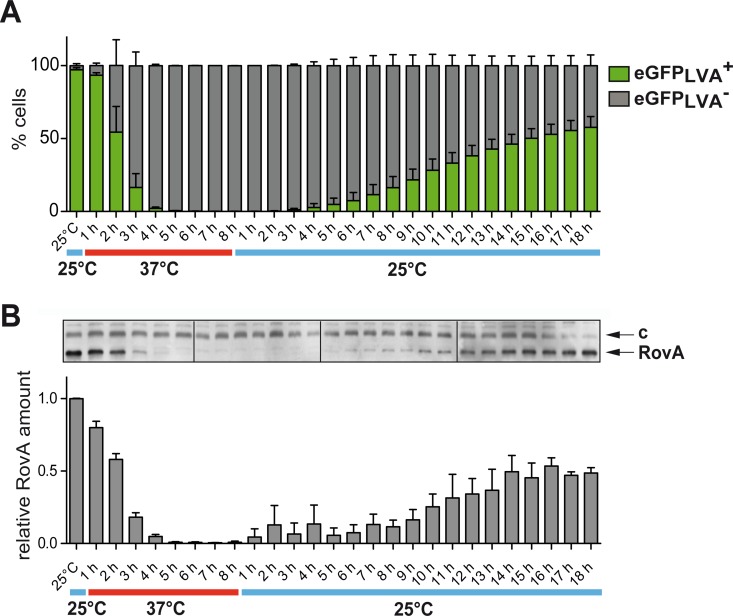
Thermal shift experiments reveal hysteresis of the temperature-responsive bistable switch. *Y*. *pseudotuberculosis* expressing P_*rovA*_-*egfp*_*LVA*_ was grown to a continuous culture in a chemostat at 25°C, shifted for 8 h to 37°C and back to 25°C for 18 h. Bacteria were analyzed by (***A***) flow cytometry (mean ± SEM; *n* = 3 for each temperature; 10^5^ cells per replicate), or (***B***) by western blot. Relative RovA amounts were quantified using ImageJ (mean ± SEM; *n* = 3 for each temperature; c: a protein band unspecifically recognized by the antiserum served as loading control).

To prove our hypothesis we first tested for the occurrence of distinct bacterial subpopulations by measuring *rovA* expression at a single-cell level. The *rovA* promoter was fused to *egfp*_*LVA*_, encoding a green fluorescence protein derivative (eGFP_LVA_) with a brighter fluorescence but reduced stability. Upon shifting from 25°C to 37°C, eGFP_LVA-_expressing *Yersinia* demonstrated successive reduction in eGFP_LVA_ synthesis, that corresponded to the average RovA level (Fig **1*B*–1*D***). Two distinct subpopulations showing no (OFF) or high (ON) eGFP_LVA_ production at growth temperatures between 30°C and 34°C were detected in the wild-type (Fig **1*B*–1*D***). No ON subpopulation could be detected when *rovA*-eGFP_LVA_ was expressed in a *rovA* mutant, confirming that expression of the reporter depends on active RovA (**S1*A*** Fig). Immunofluorescently labeled RovA-dependent adhesin InvA [16] exhibited a similar bimodal staining pattern (**S1*B* and S1*C*** Fig). Time-lapse microscopy revealed that individual bacteria can spontaneously switch (average time of 2–3 h at 32°C) from one state to the other, demonstrating reversibility of the switching process (Fig **1*E***, **S1**–**S3** Videos). We quantified the switching dynamics by measuring *rovA* expression and intracellular RovA amounts under stable physiological conditions in chemostat cultures over many generations. Quantification of bacteria in the RovA ON and OFF state after transitioning between 25°C and 37°C revealed a dependence of the system’s output on present and past inputs (hysteretic behavior) and showed that activation and deactivation of RovA synthesis occurred at strikingly different times scales (Fig **2*A* and 2*B***). Thermal upshift caused a rapid decrease in *rovA* expression with a bimodal RovA distribution and a continuous decrease in the RovA^+^ subpopulation over 3–4 h (Fig **2*A* and 2*B***). In contrast, activation of *rovA* was delayed and the RovA^+^ population increased very slowly upon thermal downshifting, indicating that the remaining amount of RovA at 37°C was insufficient to allow rapid autoinduction. In summary, this demonstrated the presence of a new, highly precise thermoresponsive bistable switch with an exceptional hysteretic behavior.

### Characterization of the temperature-responsive behavior and dynamics of the bistable switch

**Fig 3:**
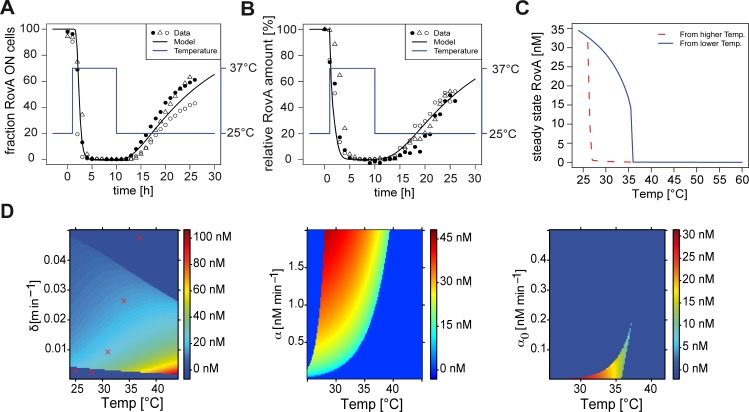
Mathematical modeling mirrors the hysteretic temperature-responsive bistable switch. (***A***) and (***B***) Stochastic model of the thermodependent dynamics of RovA bistability. The black curve represents the stochastic model, which describes the dynamics of (***A***) RovA ON cell fractions and (***B***) relative RovA amounts within the entire bacterial population. The symbols (bullet, circle, triangle) show (***A***) the fraction of RovA ON cells determined by flow cytometry from three independent temperature shift experiments over time and (***B***) quantified RovA amounts within the entire bacterial population from three independent experiments. The model reconstructs the different time scales observed in Fig **2*A***. The different symbols show The model reconstructs reliably the different time scales shown in Fig **2A** and **2B**. RovA production and the ON subpopulation are rapidly reduced upon temperature upshift as a result of the immediate inactivation and increased degradation of RovA. Net RovA production is very low at 37°C and requires more time before random fluctuations and transcriptional noise yield a critical number of active RovA molecules to reactivate its synthesis via the positive feedback loop. (***C***) Stimulus-response diagram of RovA steady-state levels in response to temperature depending on the start temperature (red 37°C, blue 25°C) demonstrates hysteresis. (***D***) A numerical approach describes the degree of bistability dependent on the degradation rate δ which is a function of temperature (left), induced production rate α (middle) or the basal production rate α_0_ (right). Red crosses represent the experimentally determined degradation rates. Bistable states are color-coded and monostable states are shown in blue.

We devised mathematical models to derive information about the underlying drivers dictating the temperature-dependent bistability of RovA (Fig **3*A***–3***C***, **S1** Text, **S2**–**S4** Figs). Our deterministic model is based on ordinary differential equations for the temporal change in RovA concentrations (d*r/*d*t*) in response to temperature (Τ) in a continuous deterministic manner. The temporal change of RovA concentration was described by a sigmoidal regulation function with a basal permanent RovA production rate α_0_ and a RovA-induced RovA production rate α. The feedback loops were coupled and influenced by an activating DNA-binding constant k_a_ and a repressive DNA-binding constant k_r_. Cooperative RovA binding was included by the Hill coefficients h_a_ and h_r_. The RovA degradation rate was included as δ. Our experimental results revealed that the DNA binding constants and the degradation rate of RovA were temperature-dependent, and thus a function of the temperature, described by Τ (k_a_(Τ), k_r_(Τ), and δ(Τ)) which leads to the resulting model:

$$\frac{dr}{dt}=\alpha_{0}+\alpha \cdot \frac{r^{h_{a}}}{k_{a}{\left(Τ\right)}^{h_{a}}+r^{h_{a}}}\frac{k_{r}{\left(Τ\right)}^{h_{r}}}{k_{r}{\left(Τ\right)}^{h_{r}}+r^{h_{r}}}-\delta (Τ)\cdot r$$

We used experimentally determined kinetic parameters to calculate the corresponding values for all temperatures and nonlinear regression to estimate the DNA-binding constants, Hill coefficients and degradation rates. To obtain the production rates α_0_ and α, we carried out stochastic modeling to fit data obtained by temperature shift experiments (**S1** Text, **S3** Fig).

A stochastic, individual-based version of the deterministic model was used to elucidate the mechanisms determining hysteresis (Fig **3*A***). The parameters obtained from chemostat experiments (α = 0.7 nM/min and α_0_ = 0.002 nM/min) predict a number of approximately 35 nM of free RovA per cell (≈25 RovA dimers), which contribute to *rovA* regulation (**S1** Text, **S3** Fig). Determination of RovA molecule numbers in *Y*. *pseudotuberculosis* expressing the P_*rovA*_-*egfp*_*LVA*_ fusion at 25°C revealed an average of 400 RovA molecules per cell, which corresponds to approximately 275 nM RovA (**S3***B* Fig). A higher concentration of RovA molecules than the predicted 35 nM is expected, since not all RovA molecules within the bacterial cell are available for autoregulation, as (i) only a fraction of RovA molecules is in the active form and (ii) a certain number of RovA dimers is also likely to be bound at different locations on the bacterial chromosome. Furthermore, the bacterial population is still in the ON state at 30°C (Fig **1*D***), while RovA amounts are considerably decreased compared to 25°C (40–50%). This indicates that less than 275 nM of RovA is sufficient to trigger RovA autoinduction in the entire population.

Our model further predicts that net production of RovA tends to be zero at 37°C as a result of the loss of DNA-binding and increased degradation rate. Consequently, RovA amounts are rapidly (within 3 h) reduced to only a few molecules. Upon downshift to 25°C, protein activation combined with the positive feedback loop can reactivate RovA synthesis, but the positive circuit is active only when sufficient RovA molecules cooperate. A simulation of the autoactivation circuit with six RovA molecules (**S1** Text) correlates perfectly with the experimental RovA data (**S3*E*** Fig). When less than six active RovA molecules are present per bacterial cell and the net production rate is very low, the population basically follows a neutral birth-death process until the critical number of active RovA molecules is produced through stochastic processes, including random fluctuations and transcriptional noise. Once this threshold is reached, the bacterial cell switches rapidly to the RovA ON state due to the positive feedback loop. Because of the time interval required to reach the critical RovA number by stochastic forces, a significantly longer time period is needed to drive the population from the RovA OFF into the RovA ON state (Figs **2** and **3*A***, **S1** Text, **S3** Fig).

A stimulus-response diagram generated by calculating the steady-state concentration of RovA at various temperatures revealed bistable response behavior with hysteresis from 27°C to 37°C highly similar to the experimental data (Fig **3*C***). The model predicted that bistability was mainly caused by the positive feedback loop, whereby the inhibitory RovA binding site reduced only the response time and degree of bistability (Fig **1*A***, **S1** Text, **S4** Fig). A numerical approach was used to describe the influence of δ, α and α_0_ on the bistable behavior (**S1** Text, Fig **3*D***). The model predicted that the thermally induced increase in degradation was crucial for temperature-responsiveness, and that α_0_ was critical for RovA bistability, whereby either extremely high or extremely low degradation and production rates abolished bistability and maintained the system in a monostable state.

**Fig 4:**
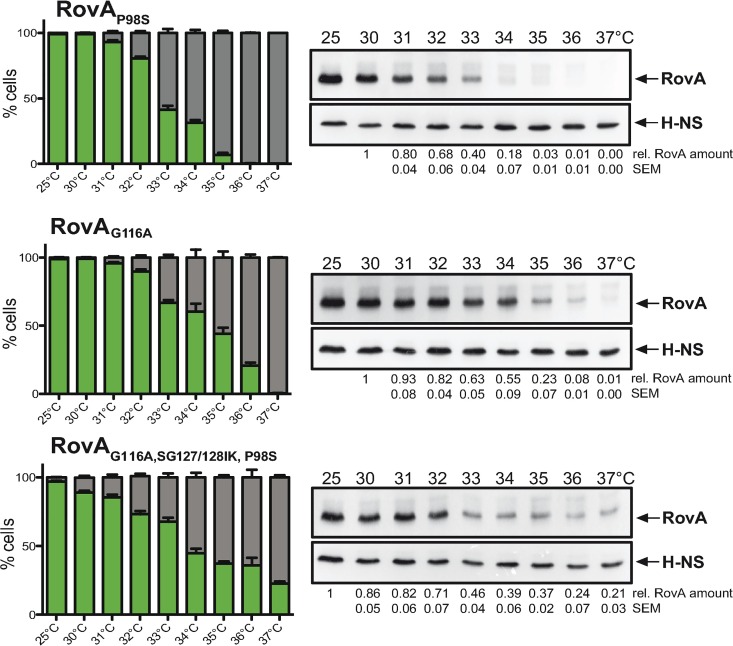
Modulation of bimodal *rovA* expression by RovA thermosensing and proteolysis. *Y*. *pseudotuberculosis rovA* mutants carrying a P_*rovA*_-*egfp*_*LVA*_ fusion were grown at different temperatures. The percentage of P_*rovA*_-*egfp*_*LVA*_-expressing wild-type and *rovA* mutant cells quantified by flow cytometry (mean ± SEM; *n* = 3 for each temperature and genotype; 10^5^ cells per replicate), eGFP_LVA_-positive cells (ON) are shown in green. The response of P_*rovA*_-*egfp*_*LVA*_ to temperature corresponds to the average RovA level as determined by western blot. Relative RovA amounts were quantified and normalised to the highest temperature for which a homogenous RovA ON population was observed (mean ± SEM; *n* = 3 for each temperature and genotype).

To challenge our analysis and mathematical predictions, we first proved whether the *rovA* regulatory region is essential for bistability. We replaced the *rovA* promoter in P_*rovA*_-*egfp*_*LVA*_ with the constitutive P_*rho*_ promoter, analyzed eGFP_LVA_ expression in *Y*. *pseudotuberculosis* strain YPIII and found that the substitution of P_*rovA*_ by P_*rho*_ eliminated bistability and resulted in a unimodal population with strong eGFP_LVA_ production from 25°C to 37°C (**S5*A***–**S5*C*** Fig). Moreover, we tested the influence of different RovA mutant proteins on bistability and found that RovA variants carrying amino acid substitutions in the thermosensing region (G116A, SG127/128IK), the Lon protease recognition site (P98S) and their combination (P98S/SG127/128IK/G116A) [5] did not abrogate bimodal *rovA* expression. The overall eGFP_LVA_ intensity of the different ON subpopulations was comparable (**S5*D*** Fig), but the temperature range for bistability was broader and shifted toward higher temperatures (Figs **1*D*** and **4**). Notably, a mutation eliminating the Lon recognition site (P98S) of RovA had no or only a very weak influence on bimodal *rovA* expression at higher temperatures. This can be explained by the fact that at 37°C the majority of RovA dimers targeted by Lon is inactive, i.e. RovA is partially defolded which abolishes its DNA-binding functions and autoactivation [4].

**Fig 5:**
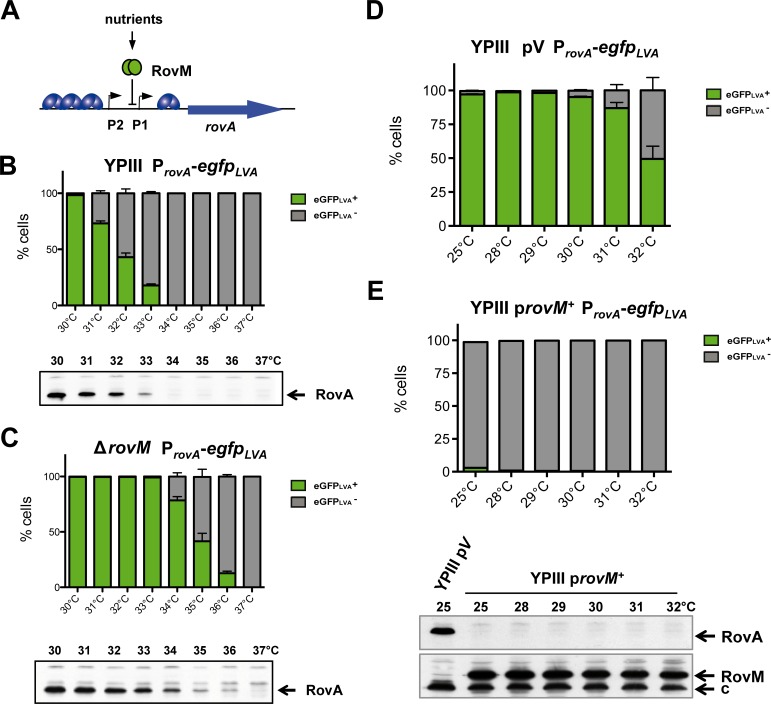
Modulation of bimodal *rovA* expression by the transcriptional repressor RovM. (***A***) The *rovA* regulatory upstream region. The RovM binding region between promoter 1 and 2 is illustrated. Binding of RovM to the *rovA* regulatory region leads to transcriptional repression of *rovA*. (***B***) *Y*. *pseudotuberculosis* YPIII wild-type, (***C***) an isogenic *rovM* deletion strain, (***D***) YPIII wild-type harboring the empty vector pV, or (***E***) the RovM overexpression vector p*rovM*^+^ carrying a P_*rovA*_-*egfp*_LVA_ fusion were grown at different temperatures. Subsequently, bacteria were fixed and analyzed by flow cytometry (mean ± SEM; *n* = 3 for each temperature; 10^5^ cells per replicate). (Below) RovA and RovM protein levels in whole cell extracts from the same cultures were analyzed by western blotting using polyclonal antibodies. An unspecific band (c) served as an internal loading control.

As shown in Fig **1*C and* 1*D*** *Yersiniae* harboring the *rovA*-eGFP_LVA_ reporter were predominantly in the OFF state at 37°C *in vitro*. However, *rovA* transcripts were identified in infected lymphatic tissues of mice by *in vivo* RNA-Seq analysis, in particular during their persistence stage in the caecum [26], indicating that additional parameters induce *rovA* transcription during infection. It is known that *rovA* expression is strongly affected by changes in carbon source availability involving the carbon storage regulator system (Csr) and the cAMP receptor protein Crp, which are transmitted through the LysR-type regulator RovM (Fig **5*A***) [17, 18]. We therefore tested whether a deletion of *rovM* influences the distribution of RovA ON and OFF cells. Strikingly, bimodal expression of *rovA* was fully preserved but shifted toward higher temperatures (Fig **5**). In the absence of RovM a significantly higher amount of RovA ON cells was observed in particular at temperatures ranging from 34°C to 36°C (Fig **5*B and* 5*C***). Moreover, a very small fraction of RovA ON cells was detectable at 25°C, but not at all tested higher temperature when RovM was overexpressed (Fig **5*D* and 5*E***).

Obviously, the observed bistable phenotype correlates with our mathematical models and appears very robust, as temperature-responsive switching was not abolished by fundamental changes in RovA stability. Moreover, the RovA ON/OFF cell ratio is adjustable by a temperature-independent regulator (RovM). This allows the pathogen to modulate the outcome in host tissues at constant temperatures according to nutrients.

### Bimodal expression of the virulence regulator RovA is crucial for virulence

**Fig 6:**
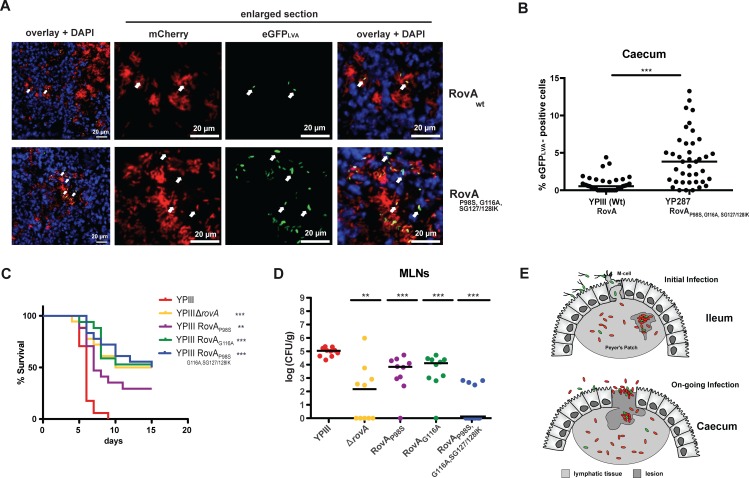
Precisely adjusted heterogeneous RovA expression is crucial for virulence. (***A***) Fluorescence microscopy of cryosections allows detection of bacteria by expression of the constitutive P_*tet*_-*mCherry* reporter (mCherry) and revealed heterogeneous expression of the P_*rovA*_-*egfp*_*LVA*_ reporter (eGFP_LVA_) in the caecum 3 days post infection. (***B***) Quantification of eGFP_LVA_-positive cells of the wild-type (YPIII) and the isogenic mutant (YP287) expressing the more stable RovA_P98S/SG127/128IK/G116A_ variant. The mean percentage of RovA-expressing bacteria was significantly higher in the mutant (***, *P* < 0.001; two-tailed Student’s *t*-test; *n* = 40 for each genotype). (***C***) Survival of mice infected with *Y*. *pseudotuberculosis* revealed reduced virulence of mutants lacking RovA or producing more stable RovA derivatives (**, *P* < 0.01, ***, *P* < 0.001; log-rank (Mantel-Cox) test; *n* = 17 for each genotype). (***D***) Infection of mice with 2 x 10^8^ bacteria either deficient in RovA or producing a more stable RovA variant led to reduced colonization of MLNs 3 days post infection (**, *P* < 0.01; ***, *P* < 0.001; two-tailed Mann-Whitney test; *n* = 10 for each genotype). (***E***) Model of bistable *rovA* expression during *Y*. *pseudotuberculosis* infection. Upon uptake from the environment, the bacteria express RovA and RovA-induced invasin, which mediates internalization into M-cells. After transcytosis into the subepithelial lymphatic tissues (Peyer’s Patches), most bacteria have switched off *rovA* expression, thereby limiting their recognition by innate immune cells, but a small RovA ON subpopulation is still found within tissue lesions. During on-going infections heterogeneity could be advantageous for persistence in the caecum as well as reinfection and spreading to other hosts when bacteria are expelled into the intestinal lumen after tissue damage.

Observed robustness of the bistable switch suggested a bimodal expression of *rovA* during infection. To obtain direct evidence for phenotypic heterogeneity *in vivo*, mice were orally challenged with *Y*. *pseudotuberculosis* using a dual fluorescence reporter system (P_tet_-*mCherry*, P_*rovA*_-*egfp*_LVA_). We observed two subpopulations in the Peyer’s patches and the caecal lymph nodes, with low numbers of RovA ON bacteria randomly distributed within microcolonies within tissue lesions (Fig **6*A* and 6*B***, **S6*A and* S6*B*** Fig). There was a statistically significant increase in the RovA ON cell population in the caecum when bacteria expressed the thermotolerant variant RovA_P98S/SG127/128IK/G116A_ (Fig **6*B***), verifying a shift of bistability towards higher temperatures *in vivo*. In contrast, no eGFP_*LVA*_-expressing bacteria were detected in the absence of the *rovA* promoter or in a *rovA* mutant strain, demonstrating that *in vivo* expression of the reporter depends on RovA (***S6C* and *S6D*** Fig).

Mice were then infected with a lethal dose (2×10^8^ bacteria) of *Y*. *pseudotuberculosis* wild-type or mutants producing the more stable RovA variants. Mice infected with wild-type bacteria displayed typical signs of the infection (e.g. weight loss, piloerection and lethargy) after 5–10 days. In contrast, none of the mice infected with a *rovA*-deficient strain or strains producing stabilized RovA variants developed severe disease symptoms, with 40–60% of the mice still alive after 15 days (Fig **6*C***). Infections with all mutants resulted in a statistically significant reduction in tissue colonization (Figs **6*D*** and **S7**). This difference was pronounced for the mesenteric lymph nodes (Fig **6*D***) from which >1000-fold fewer bacteria producing RovA_P98S/SG127/128IK/G116A_ were recovered. Smaller but statistically significant effects were observed in mice infected with mutants producing moderately stable RovA variants RovA_P98S_ and RovA_G116A_ (Fig **6*D***) indicating that variation of RovA bistable properties, which increase RovA^+^ subpopulations reduces pathogenicity. RovA^+^ cells, which express the colonization factor invasin, can efficiently invade lymphatic tissues [19–21], but its presence also renders the bacteria more susceptible to immune responses [22, 23]. A transcriptome analysis further revealed that also other surface-exposed pathogenicity factors, e.g. the afimbrial adhesin PsaA as well as lipopolysaccharide synthesis genes are activated by RovA of *Y*. *pseudotuberculosis* [24]. Although beneficial for the initiation of the infection, they are likely to trigger innate immunity-mediated antimicrobial responses when expressed in deeper tissues. In addition, several general stress adaptation genes (*ibpAB*, *uspA*, *cspB*,*C1-3*,*D*,*E*) are activated by RovA, which could support survival in the lumen of the intestine and/or in the external environment. The analysis of the RovA regulon further uncovered different metabolic programs for the wild-type and a *rovA* mutant [24], which may endow the RovA OFF population with a better fitness within lymphatic tissues. In fact, multiple enzymes of the pyruvate-TCA cycle (*icdA*, *sucDCB*, *gltA*, *acnAB*, *aceE*,*F*) are down-regulated in a *rovA* mutant, whereas several enzymes of the amino acid and nucleotide transport and metabolism are induced [24]. Different metabolic programs in the RovA ON and OFF population could contribute to the beneficial effect of bistable RovA expression as they adapt the bacterial metabolism to the distinct nutritional conditions in the intestinal tract or the lymphatic tissues. In summary, the discovered thermo-responsive bistable switch enables expression of an alternative virulence program in a small subpopulation within a single infection site. Transcriptional specialization supports survival and pathogenesis as it primes the bacteria to environmental uncertainty encountered at two critical stages when they cross the intestinal layer: (i) shortly after host entry, when *Yersinia* colonizes the intestinal tract, of which only a subset invades the Peyer’s patches [25], and (ii) during persistence in the caecum, which is a potential reservoir from which the bacteria re-emerge in the intestinal lumen after expulsion from damaged tissues (Fig **6*E***) [26, 27]. This new form of bet-hedging complements other types of heterogeneous host-pathogen interactions (i.e. slow-growing variants which are more resistant to antibiotics, or populations subsets formed within the complex tissue landscape as a response to varying local conditions faced outside and inside a bacterial microcolony [28–33]), and (ii) opposes recent approaches to targeting virulence traits such as adhesion and virulence-relevant regulatory processes to combat bacteria-mediated diseases [34–37]. Based on our study, detailed knowledge of present pathogen subsets and their distinct virulence programs including single-cell expression profiles of potential virulence targets in infected tissues are imperative for the development of successful anti-microbial therapies.

## Materials and Methods

### Bacterial strains, media and growth conditions

The strains used in this work are listed in **S1** Table. For batch culture experiments bacteria were routinely grown in Luria-Bertani (LB) broth to exponential growth phase (OD_600nm_ = 0.5–0.6) at temperatures ranging from 25°C to 37°C under aerobic conditions. If necessary, antibiotics were added at the following concentrations: carbenicillin 100 μg ml^-1^, chloramphenicol 30 μg ml^-1^ and kanamycin 50 μg ml^-1^.

### DNA manipulation and construction of plasmids

All DNA manipulations, transformations, restriction digestions and ligations were performed using standard genetic and molecular methods. The plasmids used in this work are listed in **S1** Table. Oligonucleotides used for PCR and sequencing were purchased from Metabion and are listed in **S2** Table.

Plasmid DNA was isolated using QIAprep Spin Miniprep Kit (Qiagen). DNA-modifying enzymes and restriction enzymes were purchased from Roche or New England Biolabs. PCRs were done in a 50 μl mix for 29 cycles using Phusion High-Fidelity DNA polymerase (New England Biolabs). Purification of PCR products was routinely performed using the QIAquick PCR Purfication Kit (Qiagen). All constructed plasmids were sequenced by the in-house facility.

For construction of a RovA-dependent *gfp* reporter, the *rovA* promoter region along with 170 nts of *rovA* coding region (-622 to +170) and the *egfp*_*LVA*_ gene were PCR amplified from plasmid pYPL using primers 158 and II525. The PCR product was digested with *Sal*I and *Not*I and ligated with T4-DNA ligase (NEB) into pFU76 of the pFU vector series [38], yielding plasmid pKH87. This plasmid was subsequently digested with *Sac*I and *Avr*II for the exchange of the R6K origin of replication against the origin 29807 from plasmid pFU33, resulting in plasmid pKH70.

To generate a plasmid for constitutive *egfp*_*LVA*_ expression the promoter region of the *rho* gene was PCR amplified from *Y*. *pseudotuberuclosis* YPIII genomic DNA from nucleotide -433 (primer IV490) to nucleotide -21 (primer IV491) and *egfp*_*LVA*_ was amplified with primers II525/IV483 from plasmid pKH70. The two fragments were inserted into the same backbone as pKH70 using *Aat*II and *Not*I yielding plasmid pFS5. To perform Quick-change mutagenesis (Stratagene) on *rovA*, the *rovA*^+^ plasmid pFS6 was generated. To do so, *rovA* was amplified with primers III784/III947 and ligated into *Sal*I/*Sph*I sites of the pJet1.2 cloning vector (Thermo Scientific). Quick-change mutagenesis of pFS6 was performed using primer pairs II379/II380 and II624/II625 resulting in plasmids pFS7 and pFS14, respectively. The modified versions of the *rovA* gene from pFS7 and pFS14 were transferred into the suicide mutagenesis plasmid pDM4 using *Sal*I and *Sph*I, yielding pFS8 and pFS16. Furthermore, Quick-change mutagenesis with pFS7 was performed with primers II624/II625 to generate plasmid pFS23, which was subsequently used to perform Quick-change mutagenesis with primers II626/II627 to obtain plasmid pFS24. Subsequently, pFS24 was digested with *Sal*I/*SphI* and the insert was ligated into the suicide plasmid pDM4 to obtain pFS28. For constitutive expression of P_*tet*_-*mCherry*, plasmid pFU76 was cut with *Kpn*I*/Avr*II and ligated into pZE21 resulting in plasmid pFS42. *mCherry* was amplified with primers V842/V843 from plasmid pTB23, cut with *Sal*I*/Not*I and ligated into pFS42 generating plasmid pFS43. The origin of replication p15a was amplified from plasmid pAKH120 with primers V521/V522 and cut with *Avr*II*/Sac*I. Additionally, the chloramphenicol cassette was amplified from pFU228 with primers V519/V520, cut with *Aat*II*/Sac*I and both fragments were ligated into pFS43 to obtain pFS48.

Construction of the *Y*. *pseudotuberculosis rovA* mutant strains YP269, YP270 and YP287 was performed by integration of the suicide plasmids pFS8, pFS16 or pFS28 in the *rovA* locus of strain YP107. *E*. *coli* strain S17-1λpir harbouring the plasmids were used for conjugation and the resulting transconjugants were identified by plating on *Yersinia* selective agar (Oxoid) supplemented with chloramphenicol. Expression of the *sacB* gene, which is also encoded on the integrated plasmids, is induced when the bacteria are plated on LB agar with 10% sucrose. This results in a growth reduction of the bacteria. Derivatives which have lost the plasmid due to a second recombination event were identified as more rapidly growing clones on 10% sucrose plates and presence of the individual *rovA* mutant genes was verified by PCR and sequencing with primers 135/151 as described [16].

### Flow cytometry

*Batch cultures*: *Y*. *pseudotuberculosis* YPIII harboring a RovA-dependent *egfp*_*LVA*_ reporter (pKH70) was grown over night at different temperatures ranging from 25°C to 37°C in liquid LB broth. A fresh culture was started by inoculating pre-warmed medium with over night culture in a 1:50 dilution and incubated at identical temperatures until the cultures reached an OD_600nm_ = 0.6. Subsequently, 1 ml of culture was harvested by centrifugation for western blotting and flow cytometry. For flow cytometry cell pellets were rapidly fixed in 4% para-formaldehyde for 20 min at 25°C. Pellets were washed twice with 1 x PBS and at least 100.000 cells were analyzed by a LSRII flow cytometer (BD Biosciences). Data were acquired with the FACS Diva software (BD Biosciences) and further analyzed with FlowJo v9.7.2 (Treestar).

*Continuous culture*: YPIII pKH70 was aerobically grown in Vario 500 mini-bioreactors (Medorex). The bacteria were pre-cultured for 12 h at 25°C under aerobic conditions in LB medium containing carbenicillin (100 μg ml^-1^). The bioreactor was filled with 210 ml LB medium containing carbenicillin. Antifoam 204 (Sigma-Aldrich), an entirely organic antifoaming agent, was added to the medium at a concentration of 0.02% (vol/vol). Pre-cultures were washed twice in fresh LB medium (25°C). The bioreactor was inoculated with the pre-culture (final OD_600nm_: 0.2) and run in batch mode at 25°C under continuous stirring (400 rpm). Cultivation was switched to continuous mode (25°C) at a growth rate of μ = 0.32h^-1^. After crucial processing parameters, i.e. (i) OD_600nm_: 4.6 ± 0.35; (ii) pO_2_:—45–55%; and (iii) pH 8.0 remained constant, samples were taken for western blotting and flow cytometry. Subsequently, temperature was shifted to 37°C for an 8 h period and shifted back to 25°C for additional 18 hours. Samples were taken in 1 h intervals for western blotting and flow cytometry. At least 10^5^ fixed cells were analyzed by a LSRII (BD Biosciences) flow cytometer and the data were extracted and analyzed as described above.

### Fluorescence microscopy and histology/immunofluorescence

*Batch cultures*: Poly-L-lysine solution (Sigma) was diluted 1:10 in sterile filtered 1 x PBS and spotted onto acid washed microscopy slides (VWR). After 2 hours incubation at room temperature the slides were rinsed with ultra pure water and air dried over night. In 4% para-formaldehyde fixed *Yersinia* cell suspensions (OD_600nm_ 0.6) were diluted 1:10 in 1 x PBS. This dilution was spotted onto the poly-L-lysine-coated microscopy slides and incubated for 30 min at room temperature. The slides were washed 3 times with 1 x PBS and cells were blocked for 1 h in 1 x PBS containing 2% BSA. For immunostaining of invasin (InvA), slides were then washed with 1 x PBS before addition of the anti-InvA^42^ monoclonal mouse IgG (1:1.000 in 1 x PBS containing 1% BSA). After 1 h incubation at room temperature the slides were washed 3 times with 1 x PBS. The secondary antibody (goat anti-mouse IgG, Cy5 conjugate, Invitrogen) was added in a 1:1.000 dilution and slides were incubated for an additional hour at room temperature. Slides were washed 3 times in 1 x PBS. Coverslips were mounted using SlowFade Gold (Life Technologies), covered with a glass slide and analysed with an Axiovert II fluorescence microscope (Zeiss) with an Axiocam HR digital charge-coupled device (CCD) camera (Zeiss) and the AxioVision program (Zeiss) and the software ImageJ (https://imagej.nih.gov/ij/).

*Infected tissue*: *Y*. *pseudotuberculosis* YPIII and YP287 harboring a P_*rovA*_::*egfp*_*LVA*_ fusion (pKH70) and a P_*tet*_::*mCherry* expression construct (pFS48) as well as *Y*. *pseudotuberculosis* YPIII harboring only pFS48, which served as negative control, were grown in LB medium at 25°C overnight. Mice were infected orally with 2 x 10^8^ bacteria. After three days mice were sacrificed by CO_2_ asphyxiation. For cryosections, the Peyer’s patches and caeca were frozen in Tissue-Tek OCT freezing medium (Sakura Finetek) on dry ice. Sections of 6–10 μm were prepared using a Microm HM 560 cryostat (Thermo Scientific) and mounted on SuperFrost Plus slides (Thermo Scientific). Air-dried sections were fixed for 20 min in ice-cold 4% para-formaldehyde and washed twice with PBS. For visualization of nuclei in the fixed tissue, samples were stained and mounted with Roti Mount Flour Core 49,6-diamidino-2-phenylindole (DAPI, Roth). Tissues were imaged and localization of *Yersiniae* in the infected tissues was analyzed using a fluorescence microscope (Axiovert II, Zeiss) with 25 x and 40 x objectives, an Axiocam HR digital charge-coupled device (CCD) camera (Zeiss) and the ZEN program (Zeiss). Total number of mCherry-positive bacteria and the number of the cells expressing also eGFP_LVA_ was counted in 40 randomly chosen tissue sections of the Peyer’s patches and the caecum of three infected mice, and the percentage of cells expressing eGFP_LVA_ was calculated.

### Time-lapse fluorescence microscopy

For time-lapse microscopy bacteria were grown over night in LB medium at 32°C in the presence of carbenicillin. A fresh culture was started by inoculating pre-warmed LB medium with an over night culture in a 1:50 dilution and was grown at the same temperature to OD_600nm_ of 0.6. Subsequently, 1 μl of bacterial culture was distributed on a microdish (IBIDI), overlaid with a thin agarblock (2% LB-agar with carbenicillin) and covered with a glass slide.

For live cell imaging, shutters were computer-controlled, synchronized with the HR camera and opened only during exposure time to reduce photobleaching of eGFP_LVA_ and photodamage of the cells. Starting from single cells or cell doublets, eGFP_LVA_ fluorescence was recorded over several generations. Each imaging cycle consisted of one fluorescence frame to track eGFP_LVA_ expression, followed by one phase-contrast frame to monitor also those cells, which do not express eGFP_LVA_. The temperature of the microscope chamber was controlled by the Heating Unit XL S and the Incubator XL S (Zeiss). A stable focus was ensured over several hours of imaging by using the Definite-Focus system (Zeiss). Captured images were processed using the Axiovision or ZEN software (Zeiss) and the software ImageJ (https://imagej.nih.gov/ij/).

### Western blotting

For the detection of RovA, RovM and H-NS, bacterial whole cell extracts were prepared from equal amounts of bacteria and separated on SDS-polyacrylamide gels, and blotted onto nitrocellulose membranes. Subsequently, membranes were blocked in 1 x TBST containing 3% BSA (blocking buffer). Primary anti-RovA [4], anti-RovM [17] and anti-H-NS [15] antibodies were added in a 1:4.000 dilution in blocking buffer. The secondary antibody, anti-rabbit IgG conjugated with horseradish peroxidase, was supplied in a 1:8.000 dilution in blocking buffer and the immunological detection of the proteins was performed as described previously[15, 17].

### Purification of recombinant *Y*. *pseudotuberculosis* RovA

His-tagged RovA was overexpressed with BL21λDE3 pLW2 and purified as described earlier [4].

### Electrophoretic mobility shift assays (EMSAs)

EMSAs were performed as described [4]. The DNA fragments of the *rovA* regulatory regions including either RovA binding site I or II were amplified with the primer pairs 153/296 and 178/V99. For competitive EMSAs DNA fragments containing either RovA binding site I or II were mixed in equimolar amounts. Pre-incubation of recombinant RovA with the DNA fragments and native gel electrophoresis were performed at 25°C and 37°C, respectively.

### Determination of the RovA molecule number per cell

To determine the amount of RovA molecules per cell *Y*. *pseudotuberculosis* YPIII harbouring a RovA-dependent *egfp*_*LVA*_ reporter (pKH70) was grown over night at 25°C in liquid LB broth. A fresh culture was started by inoculating pre-warmed medium with over night culture in a 1:50 dilution and incubated at identical temperatures until the cultures reached an OD_600nm_ = 0.6. Subsequently, 1 ml of culture was harvested by centrifugation for western blotting. The bacterial pellet was resuspended in 60 μl 1 x SDS loading dye, heated to 95°C for 10 min, cooled on ice and centrifuged for 5 min at 10.000 g. 10 μl of supernatant (bacterial cell extract from approx. 10^8^ bacteria) were loaded onto 15% polyacrylamide SDS gels. In parallel 1 and 3 ng of recombinant RovA were loaded. Western blotting was performed as described.

### Mouse infection

For survival and organ burden experiments, 6–7 week old female Balb/c mice were purchased from Janvier (Saint Berthevin Cedex, France) and housed under specific pathogen-free conditions in the animal facility of the Helmholtz Centre for Infection Research, Braunschweig. After 16 hours of starvation, mice were orally infected with approximately 2 x 10^8^ colony forming units (cfu) of *Y*. *pseudotuberculosis* YPIII or the different isogenic *rovA* mutant strains using a gavage needle. Bacteria were grown over night in LB medium at 25°C, washed and resuspended in PBS. For survival experiments infected mice were monitored for 14 days on a daily basis to determine the survival rate, the body weight and health status. For organ burden experiments, mice were euthanized by CO_2_ asphyxiation three days after infection. Peyer’s patches, caecum, MLNs, liver and spleen were isolated. Subsequently, all organs were weighed and homogenized in PBS at 30.000 rpm for 30 sec using a Polytron PT 2100 homogenizer (Kinematica, Switzerland). To determine the bacterial load of the organs serial dilutions of the homogenates were plated on LB plates with triclosan (Calbiochem). The cfu were counted and are given as cfu per g organ/tissue. To assure presence of the reporter plasmids during infection serial dilutions of Peyer’s patches and caecum of four infected mice were plated in parallel on LB plates containing either triclosan (total bacteria) or a combination of triclosan, chloramphenicol and carbenicillin. The cfu were counted and are given as percentage of cfu, normalized to the amount of total bacteria.

### Ethics statement

Animal housing and all animal experiments were performed in strict accordance with the German Recommendations of the Society of Laboratory Animal Science (GV-SOLAS) and the European Health Recommendations of the Federation of Laboratory Animal Science Associations (FELASA). The animal care and use protocols adhered to the German Animal Welfare Act, Tierschutzgesetz (TierSchG) and were approved by the Niedersächsisches Landesamt für Verbraucherschutz und Lebensmittelsicherheit: animal licensing committee permission no. 33.9.42502-04-12/1010. Animals were handled with appropriate care and all efforts were made to minimize suffering.

### Statistics

Statistical tests were performed with Prism 5.0c (GraphPad Software). Mann-Whitney test was used to compare wild-type and the *rovA* mutants in the organ burden experiments. The survival was statistical analyzed by the log-rank (Mantel-Cox) test. The amount of green bacteria in microcolonies in the infected tissues was compared between wild-type and the *rovA* mutant using Students t-test. p values < 0.05 were considered significant.

## Supporting Information

S1 Fig eGFP_LVA_ expression is RovA-dependent and correlates with InvA amount on the cell surface. (***A***) *Y*. *pseudotuberculosis* YPIII *rovA* deletion mutant carrying a P_*rovA*_-*egfp*_*LVA*_ fusion was grown at different temperatures, fixed and analyzed by flow cytometry (*n* = 3 for each temperature; 10^5^ cells per replicate). Numbers of P_*rovA*_-*egfp*_*LVA*_-expressing cells are illustrated in percentage. eGFP_LVA_ -positive cells (ON state) are given in green. (***B***) Expression of the P_*rovA*_-*egfp*_*LVA*_ reporter of *Y*. *pseudotuberculosis* is bistable at 32°C. At 32°C, bacteria which are in the RovA ON state and express eGFP_LVA_ in a RovA-dependent manner are supposed to carry significantly more invasin on the cell surface compared to cells which are in the OFF state and do not express eGFP_LVA_. (***C***) *Y*. *pseudotuberculosis* YPIII and an isogenic *invA* mutant (YPIII Δ*invA*) carrying a P_*rovA*_-*egfp*_*LVA*_ fusion were grown at 32°C and/or 25°C (*n* = 3), fixed and stained with a monoclonal InvA IgG (secondary AB: goat anti-mouse IgG, Cy5 conjugate). Representative microscopic images are illustrated.
(JPG)

S2 FigModeling of the temperature-responsive behaviour of RovA.(**A**-**C**) RovA degradation rates at various temperatures. (***A***) Cultures of *Y*. *pseudotuberculosis* strain YPIII were grown to exponential phase (OD_600_ = 0.3–0.4) at 25°C before chloramphenicol (200 μg ml^**-**1^) was added. The cultures were divided and incubated at 25°C, 28°C, 31°C, 34°C or 37°C for additional 90 min. Aliquots of the cultures were removed at the indicated times, whole cell extracts from identical numbers of bacteria were prepared and analyzed by western blotting with a polyclonal antibody directed against RovA. (***B***) For each temperature, a point estimate of the degradation rate δ was determined. (***C***) A non-linear regression with the function $\delta (Τ)=\delta_{0}\cdot e^{-\delta_{1}Τ}$ was performed resulting in the function (8) for the temperature-dependent degradation rate. (***D****-****H***) Temperature-dependent DNA binding constants of RovA. (***D***) Increasing concentrations of purified RovA were incubated with *rovA* promoter fragments harboring the activating RovA binding site (BS I) or the repressing RovA binding site (BS II) at 25°C or 37°C. The resulting DNA-protein complexes were separated on 4% polyacrylamide gels and the bands of the free (unshifted) *rovA* promoter fragments were quantified using ImageJ [39] (mean ± SEM; *n* = 3). The percentage of unshifted *rovA* promoter fragments BS I and BS II is given relative to the promoter bands in the absence of RovA defined as 100%. Titration of increasing RovA concentrations allowed quantification of RovA binding (round symbols) and fitting of the binding constants k_d_ (half-saturation constants) according to equation (9) (black line) is given in panel ***E****-****H***. The binding constant k_a_ for the activating binding site is shown in (***E***) for 25^◦^C and in (***F***) for 37^◦^C, the binding site k_r_ for the repressing binding site is shown in (***G***) for 25^◦^C and in (**H)** for 37^◦^C. (***I*, *J***) Temperature dependency of the DNA-binding constants of RovA. The RovA DNA binding constants **I**, k_a_ for the activating binding site and **J**, k_r_ for the repressing binding site were determined by non-linear regression with integer Hill coefficients. The temperature dependence of the binding constants was extrapolated using exponential functions (10) (black line) to avoid negative values at low temperatures which would result from a linear fit (dashed line).(JPG)

S3 Fig Modeling of RovA dynamics in response to thermal shifts. (***A***) Estimation of the RovA production rate. Contour plot of fitting error over α and α_0_. The optimal parameter values for α and α_0_ are indicated by a bullet. (***B***) Determination of RovA molecules per cell at 25°C. *Y*. *pseudotuberculosis* wild-type strain YPIII expressing the P_*rovA*_-*egfp*_*LVA*_ fusion was grown at 25°C. Cell extracts of two separate cultures of 10^8^ bacteria were prepared, separated on SDS-polyacrylamide gels together with 1.0 and 3.0 ng of recombinant His-tagged RovA protein (RovA-His_6_) and subjected to western blotting using a polyclonal antibody against RovA. The protein bands were quantified (*n* = 2) and used to calculate the number of RovA molecules in the bacterial cell (see also part 2: stochastic model, **S1** Text). (***C***) Quantities of RovA expressing cells at four selected time points (12, 16, 21 and 25 h) after the start of the temperature shift experiment are accurately mirrored by the mathematical modeling approach. The lower histogram in each quadrant represents the experimental data and the upper histogram the results of the stochastic model. (***D-E***) Modeling of the extended lag phase of RovA production following a thermal downshift. Based on the experimental data it is proposed that the RovA population follows a neutral stochastic birth-death process. For modeling, a basic random walk on 0,…,*N* RovA molecules was considered as described in the Supplementary equations 12–14. (***D***) Transition graph of the model. (***E***) A simulation of the fraction of RovA ON cells with *N* = 6 (black line) correlates perfectly with the experimental data (symbols: bullet, circle, triangle represent the independent experiments; *n* = 3).
(JPG)

S4 Fig Analysis of RovA bistability without the inhibitory RovA binding site. (***A***) Levelplots show the degree of bistability in dependence on the degradation rate δ (left), the induced production rate α (middle), and the basal production rate α_0_ (right) at different temperatures. Red crosses illustrate the experimentally determined degradation rates. Bistable states are color-coded and monostable states are indicated in blue. (***B***) Stimulus-response diagram of RovA steady state levels of the regulatory system without the inhibitory binding site in response to a temperature shift (red: shift from 37°C to 25°C, blue: shift from 25°C to 37°C) leads to alterations in the degree of bistability and changes the robustness of stable states. (***C*, *D***) Response time of the RovA regulatory system to temperature shifts. Analysis of the response time of the regulatory system with and without the inhibitory binding site in response to a temperature shift (***C***) from 25°C to higher temperatures or (***D***) from 37°C to lower temperatures demonstrated that presence of the inhibitory site prolongs the response time in particular to a thermal upshift. Blue line: wild-type system, red line: without inhibitory RovA binding site.
(JPG)

S5 Fig Bimodal expression of P*_rovA_*-*egfp_LVA_* fusion is specific to RovA. (***A***) The *egfp*_*LVA*_ reporter fusions flanked by the *rovA* and *rho* regulatory upstream region, respectively. (***B***) *Y*. *pseudotuberculosis* YPIII wild-type strain carrying a P_*rho*_-*egfp*_*LVA*_ fusion was grown at different temperatures. Subsequently bacteria were fixed and analyzed by flow cytometry (one representative replicate is shown; 10^5^ cells). (***C***) Numbers of P_*rho*_-*egfp*_*LVA*_-expressing cells are illustrated in percentage (mean ± SEM; *n* = 3 for each temperature; 10^5^ cells per replicate). eGFP_LVA_-positive cells (ON state) are given in green. (***D***) *Y*. *pseudotuberculosis* YPIII wild-type and *rovA* mutant strains carrying a P_*rovA*_-*egfp*_*LVA*_ fusion were grown at 32°C, fixed and analyzed by flow cytometry (one representative replicate is shown; 10^5^ cells).
(JPG)

S6 Fig Infection experiments demonstrate heterogeneity of RovA expression in the Peyer’s patches. Fluorescence microscopy of cryosections of Peyer’s Patches (PPs) of female Balb/c mice 3 days post infection with *Y*. *pseudotuberculosis* (***A***) expressing the RovA wild-type protein or (***B***) the stable RovA_P98S/SG127/128IK/G116A_ variant. The entire bacterial population within one cryosection was detected by expression of a constitutive P_*tet*_-*mCherry* reporter (mCherry). Fluorescence microscopy revealed heterogeneous expression of the P_*rovA*_-e*gfp*_*LVA*_ reporter (eGFP_LVA_) in the presence of RovA (YPIII) or its stable variant (YP287), while no eGFP_LVA_-positive cells were detected in the absence of (***C***) P_*rovA*_-*egfp*_*LVA*_ or **(*D*)** RovA_._ **(*E*)** Bacteria from infected tissues were plated on LB-agar with or without respective antibiotics (mean ± SEM; *n* = 4 for each genotype and tissue) to assure presence of the reporters during infection.
(JPG)

S7 Fig Heterogeneous RovA expression is crucial for virulence. Oral infection of female Balb/c mice with 2 x 10^8^ bacteria producing wild-type RovA (YPIII), no RovA (Δ*rovA*) or more stable RovA variants (RovA_P98S_, RovA_G116A_, RovA_P98S,G116A,SG127/128IK_) led to reduced colonization of Peyer’s patches, caecum and spleen 3 days post infection (**, p < 0.01; *, p < 0.05; two-tailed Mann-Whitney test; *n* = 10 for each genotype and tissue).
(JPG)

S1 Table Bacterial strains and plasmids. This table lists and describes all plasmids and strain used in this study, including their sources and references.
(DOCX)

S2 Table Oligonucleotides. This table lists all primers and their sequences used in this study. The corresponding restriction sites are underlined. Highlighted in bold are nucleotides exchanged by Quick-Change mutagenesis of *rovA*.
(DOCX)

S3 Table Temperature-dependent DNA binding constants of RovA. Calculated DNA binding constants for the activating and repressive RovA binding site at 25°C and 37°C are presented with open and fixed Hill coefficients.
(PDF)

S4 Table Model parameters. All parameters used in the deterministic model (left column) and the stochastic model (right column) describing the shift experiment are listed.
(PDF)

S1 Text Mathematical modeling approaches. This section includes the mathematical modelling approaches to simulate temperature-dependent bistability of the molecular thermometer RovA and the dynamics of the thermoresponsive bistable switch. It describes the determination of the parameters chosen for the simulation and includes a discussion of the models along with the experimental data of this study.
(DOCX)

S1 Video Live cell imaging of *Y. pseudotuberculosis* expressing P*_rovA_*-*egfp_LVA_* by fluorescence microscopy (40 x oil-immersion objective) at 32°C. In 12 min intervals a fluorescence frame was taken to track P_*rovA*_-*egfp*_*LVA*_ expression, followed by a phase-contrast frame. The videos demonstrate spontaneous switching from eGFP_LVA_ expressing (ON) into eGFP_LVA_ non-expressing cells (OFF) and vice versa. Growth of a cell starting in the OFF state (588 min).
(MOV)

S2 Video Live cell imaging of *Y. pseudotuberculosis* expressing P*_rovA_*-*egfp_LVA_* by fluorescence microscopy (40 x oil-immersion objective) at 32°C. In 12 min intervals a fluorescence frame was taken to track P_*rovA*_-*egfp*_*LVA*_ expression, followed by a phase-contrast frame. The videos demonstrate spontaneous switching from eGFP_LVA_ expressing (ON) into eGFP_LVA_ non-expressing cells (OFF) and vice versa. Three cells starting from the OFF and one from the ON state (360 min).
(MOV)

S3 Video Live cell imaging of *Y. pseudotuberculosis* expressing P*_rovA_*-*egfp_LVA_* by fluorescence microscopy (40 x oil-immersion objective) at 32°C. In 12 min intervals a fluorescence frame was taken to track P_*rovA*_-*egfp*_*LVA*_ expression, followed by a phase-contrast frame. The videos demonstrate spontaneous switching from eGFP_LVA_ expressing (ON) into eGFP_LVA_ non-expressing cells (OFF) and vice versa. Growth of a cell starting in the OFF state (588 min). Three cell doublets starting in the OFF state (348 min).
(MOV)
