# Supplementary figures and images for: A Precise Temperature-Responsive Bistable Switch Controlling Yersinia Virulence
Source: PLoS Pathog. 2016 Dec 22;12(12):e1006091. doi: 10.1371/journal.ppat.1006091 (PMC5179001; doi:10.1371/journal.ppat.1006091)

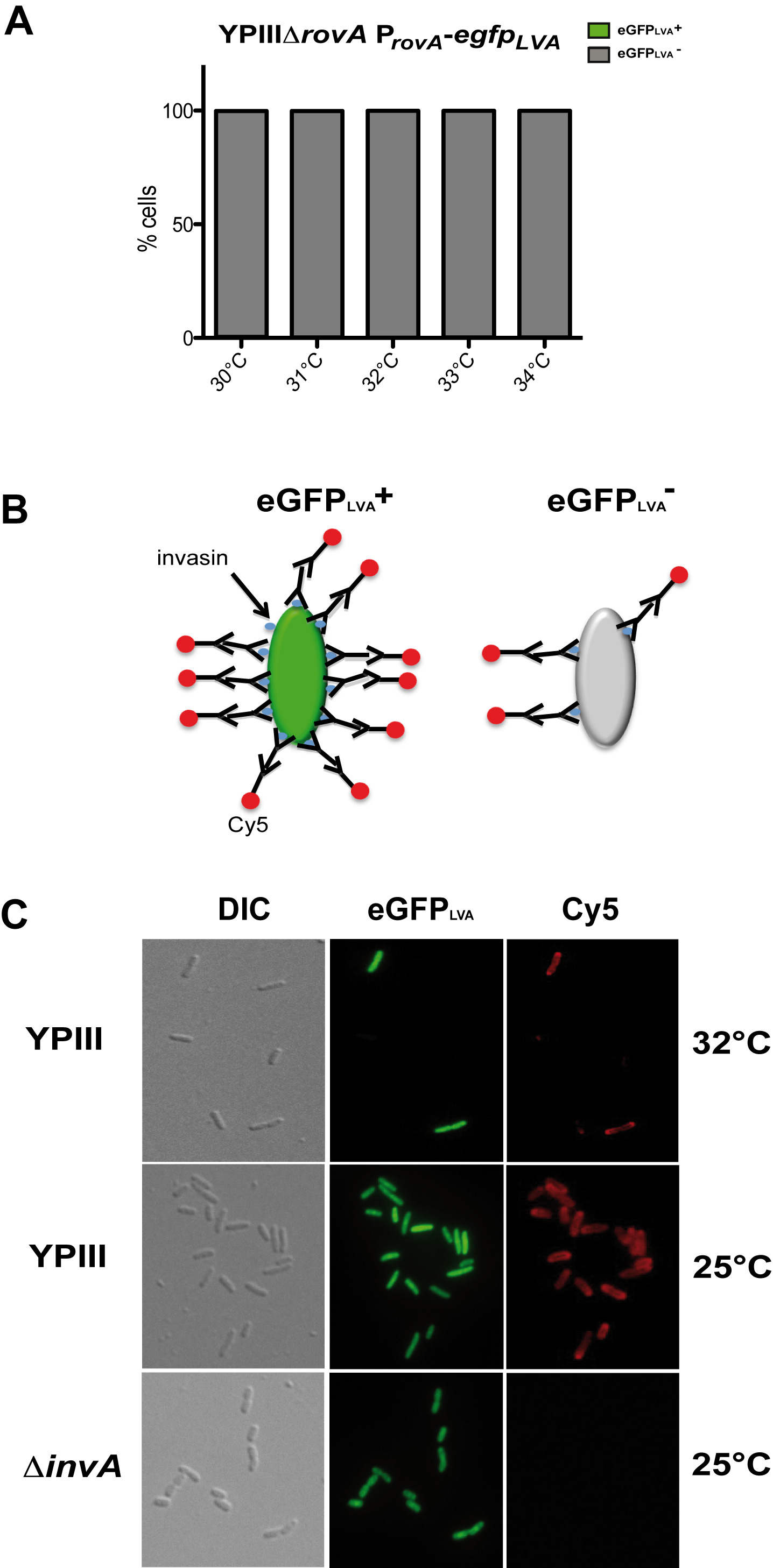

Supplement: S1 Fig — (A) Y. pseudotuberculosis YPIII rovA deletion mutant carrying a ProvA-egfpLVA fusion was grown at different temperatures, fixed and analyzed by flow cytometry (n = 3 for each temperature; 105 cells per replicate). Numbers of ProvA-egfpLVA-expressing cells are illustrated in percentage. eGFPLVA -positive cells (ON state) are given in green. (B) Expression of the ProvA-egfpLVA reporter of Y. pseudotuberculosis is bistable at 32°C. At 32°C, bacteria which are in the RovA ON state and express eGFPLVA in a RovA-dependent manner are supposed to carry significantly more invasin on the cell surface compared to cells which are in the OFF state and do not express eGFPLVA. (C) Y. pseudotuberculosis YPIII and an isogenic invA mutant (YPIII ΔinvA) carrying a ProvA-egfpLVA fusion were grown at 32°C and/or 25°C (n = 3), fixed and stained with a monoclonal InvA IgG (secondary AB: goat anti-mouse IgG, Cy5 conjugate). Representative microscopic images are illustrated. (JPG) [file ppat.1006091.s001.jpg]

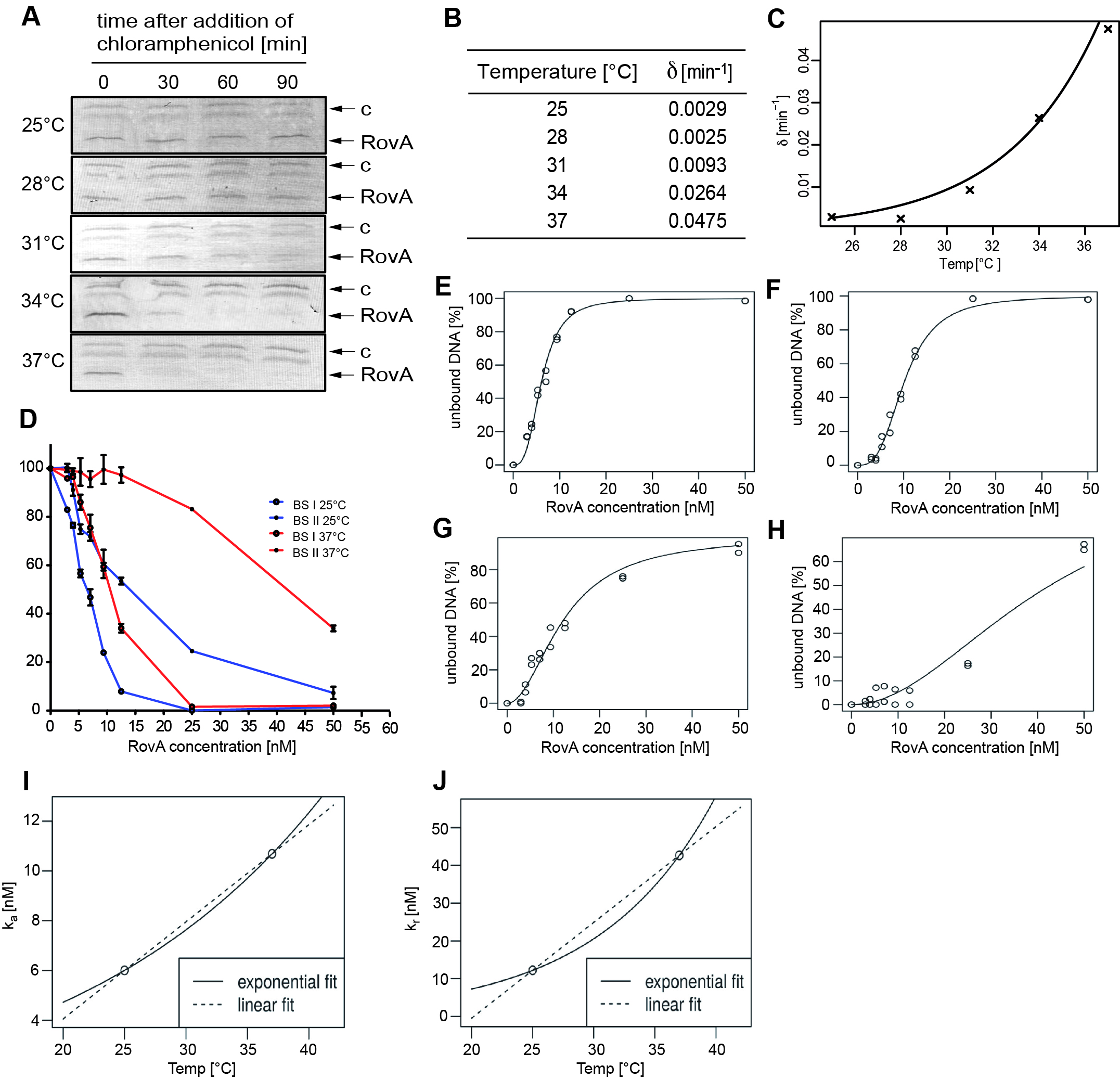

Supplement: S2 Fig — (A-C) RovA degradation rates at various temperatures. (A) Cultures of Y. pseudotuberculosis strain YPIII were grown to exponential phase (OD600 = 0.3–0.4) at 25°C before chloramphenicol (200 μg ml-1) was added. The cultures were divided and incubated at 25°C, 28°C, 31°C, 34°C or 37°C for additional 90 min. Aliquots of the cultures were removed at the indicated times, whole cell extracts from identical numbers of bacteria were prepared and analyzed by western blotting with a polyclonal antibody directed against RovA. (B) For each temperature, a point estimate of the degradation rate δ was determined. (C) A non-linear regression with the function δ(Τ)=δ0⋅e−δ1Τ was performed resulting in the function (8) for the temperature-dependent degradation rate. (D-H) Temperature-dependent DNA binding constants of RovA. (D) Increasing concentrations of purified RovA were incubated with rovA promoter fragments harboring the activating RovA binding site (BS I) or the repressing RovA binding site (BS II) at 25°C or 37°C. The resulting DNA-protein complexes were separated on 4% polyacrylamide gels and the bands of the free (unshifted) rovA promoter fragments were quantified using ImageJ [39] (mean ± SEM; n = 3). The percentage of unshifted rovA promoter fragments BS I and BS II is given relative to the promoter bands in the absence of RovA defined as 100%. Titration of increasing RovA concentrations allowed quantification of RovA binding (round symbols) and fitting of the binding constants kd (half-saturation constants) according to equation (9) (black line) is given in panel E-H. The binding constant ka for the activating binding site is shown in (E) for 25◦C and in (F) for 37◦C, the binding site kr for the repressing binding site is shown in (G) for 25◦C and in (H) for 37◦C. (I, J) Temperature dependency of the DNA-binding constants of RovA. The RovA DNA binding constants I, ka for the activating binding site and J, kr for the repressing binding site were determined by non-linear r [file ppat.1006091.s002.jpg]

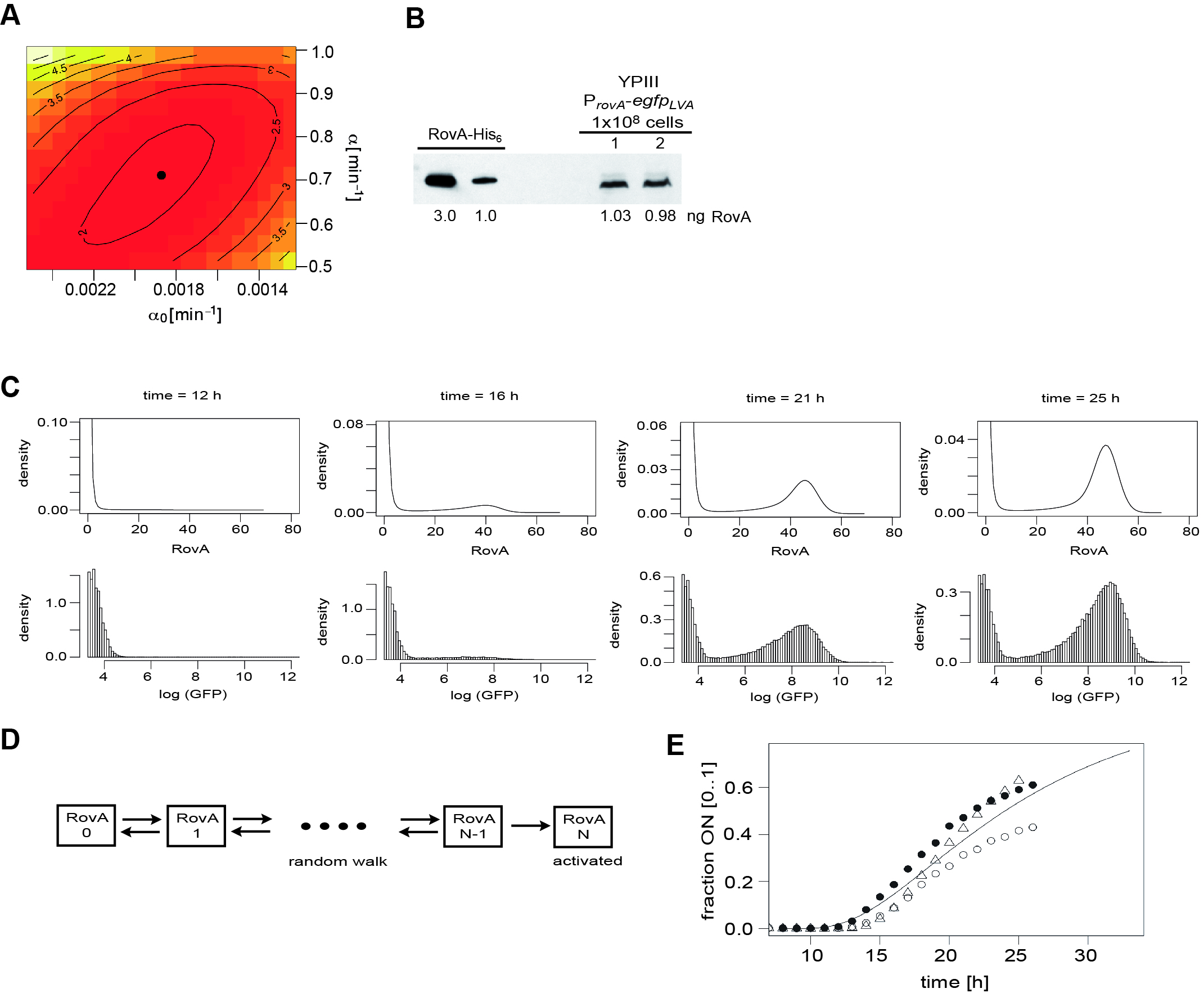

Supplement: S3 Fig — (A) Estimation of the RovA production rate. Contour plot of fitting error over α and α0. The optimal parameter values for α and α0 are indicated by a bullet. (B) Determination of RovA molecules per cell at 25°C. Y. pseudotuberculosis wild-type strain YPIII expressing the ProvA-egfpLVA fusion was grown at 25°C. Cell extracts of two separate cultures of 108 bacteria were prepared, separated on SDS-polyacrylamide gels together with 1.0 and 3.0 ng of recombinant His-tagged RovA protein (RovA-His6) and subjected to western blotting using a polyclonal antibody against RovA. The protein bands were quantified (n = 2) and used to calculate the number of RovA molecules in the bacterial cell (see also part 2: stochastic model, S1 Text). (C) Quantities of RovA expressing cells at four selected time points (12, 16, 21 and 25 h) after the start of the temperature shift experiment are accurately mirrored by the mathematical modeling approach. The lower histogram in each quadrant represents the experimental data and the upper histogram the results of the stochastic model. (D-E) Modeling of the extended lag phase of RovA production following a thermal downshift. Based on the experimental data it is proposed that the RovA population follows a neutral stochastic birth-death process. For modeling, a basic random walk on 0,…,N RovA molecules was considered as described in the Supplementary equations 12–14. (D) Transition graph of the model. (E) A simulation of the fraction of RovA ON cells with N = 6 (black line) correlates perfectly with the experimental data (symbols: bullet, circle, triangle represent the independent experiments; n = 3). (JPG) [file ppat.1006091.s003.jpg]

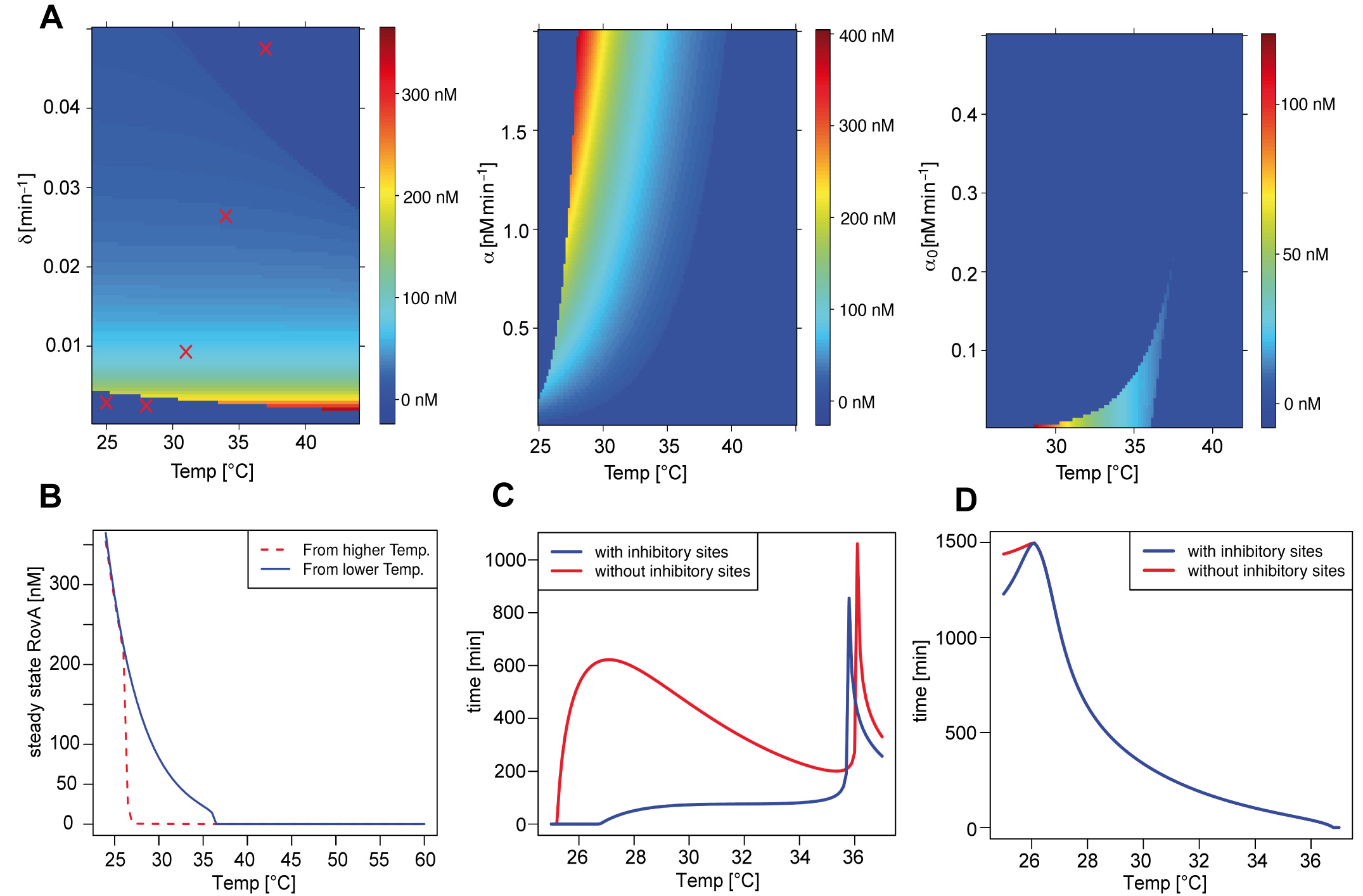

Supplement: S4 Fig — (A) Levelplots show the degree of bistability in dependence on the degradation rate δ (left), the induced production rate α (middle), and the basal production rate α0 (right) at different temperatures. Red crosses illustrate the experimentally determined degradation rates. Bistable states are color-coded and monostable states are indicated in blue. (B) Stimulus-response diagram of RovA steady state levels of the regulatory system without the inhibitory binding site in response to a temperature shift (red: shift from 37°C to 25°C, blue: shift from 25°C to 37°C) leads to alterations in the degree of bistability and changes the robustness of stable states. (C, D) Response time of the RovA regulatory system to temperature shifts. Analysis of the response time of the regulatory system with and without the inhibitory binding site in response to a temperature shift (C) from 25°C to higher temperatures or (D) from 37°C to lower temperatures demonstrated that presence of the inhibitory site prolongs the response time in particular to a thermal upshift. Blue line: wild-type system, red line: without inhibitory RovA binding site. (JPG) [file ppat.1006091.s004.jpg]

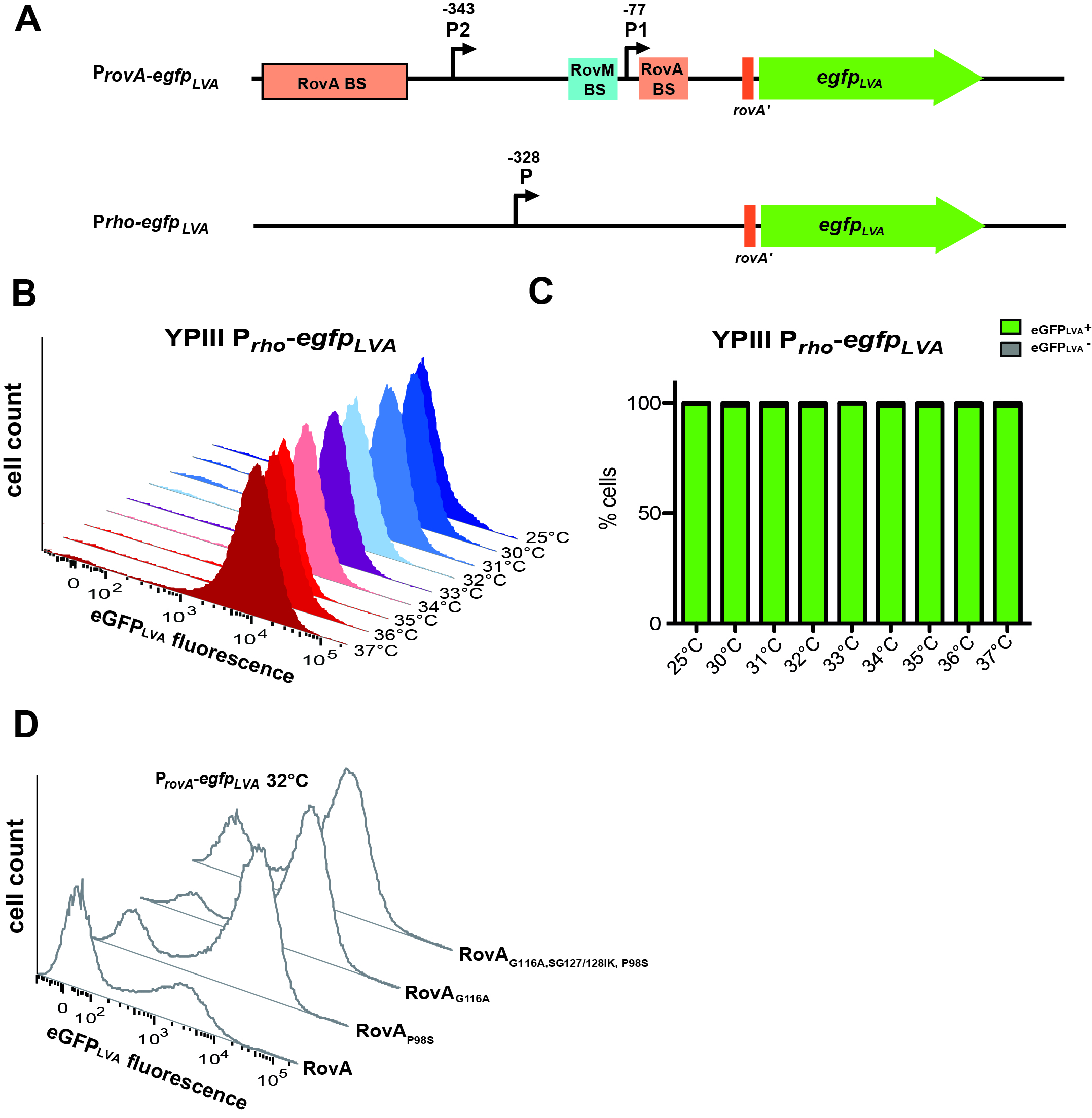

Supplement: S5 Fig — (A) The egfpLVA reporter fusions flanked by the rovA and rho regulatory upstream region, respectively. (B) Y. pseudotuberculosis YPIII wild-type strain carrying a Prho-egfpLVA fusion was grown at different temperatures. Subsequently bacteria were fixed and analyzed by flow cytometry (one representative replicate is shown; 105 cells). (C) Numbers of Prho-egfpLVA-expressing cells are illustrated in percentage (mean ± SEM; n = 3 for each temperature; 105 cells per replicate). eGFPLVA-positive cells (ON state) are given in green. (D) Y. pseudotuberculosis YPIII wild-type and rovA mutant strains carrying a ProvA-egfpLVA fusion were grown at 32°C, fixed and analyzed by flow cytometry (one representative replicate is shown; 105 cells). (JPG) [file ppat.1006091.s005.jpg]

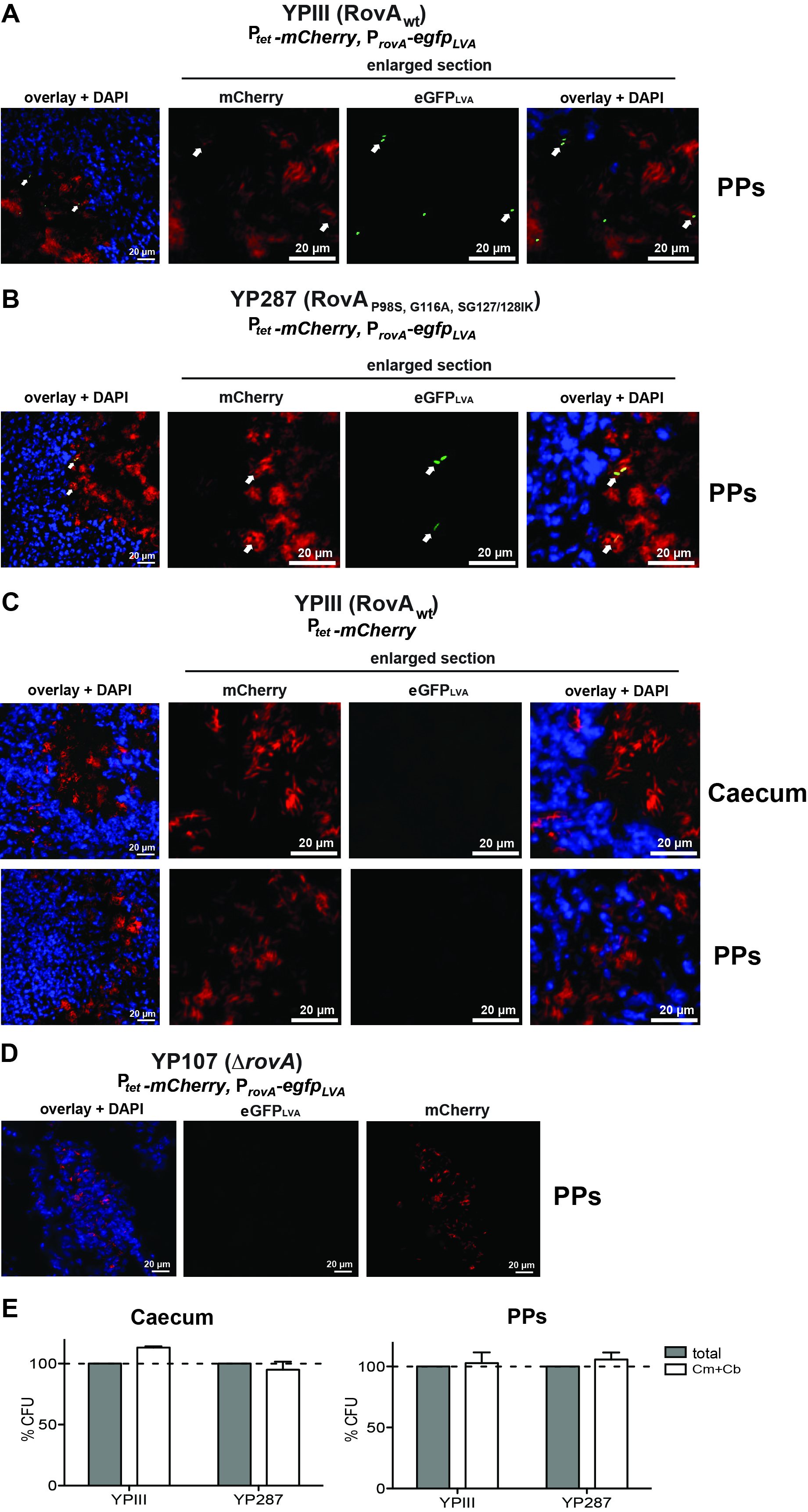

Supplement: S6 Fig — Fluorescence microscopy of cryosections of Peyer’s Patches (PPs) of female Balb/c mice 3 days post infection with Y. pseudotuberculosis (A) expressing the RovA wild-type protein or (B) the stable RovAP98S/SG127/128IK/G116A variant. The entire bacterial population within one cryosection was detected by expression of a constitutive Ptet-mCherry reporter (mCherry). Fluorescence microscopy revealed heterogeneous expression of the ProvA-egfpLVA reporter (eGFPLVA) in the presence of RovA (YPIII) or its stable variant (YP287), while no eGFPLVA-positive cells were detected in the absence of (C) ProvA-egfpLVA or (D) RovA. (E) Bacteria from infected tissues were plated on LB-agar with or without respective antibiotics (mean ± SEM; n = 4 for each genotype and tissue) to assure presence of the reporters during infection. (JPG) [file ppat.1006091.s006.jpg]

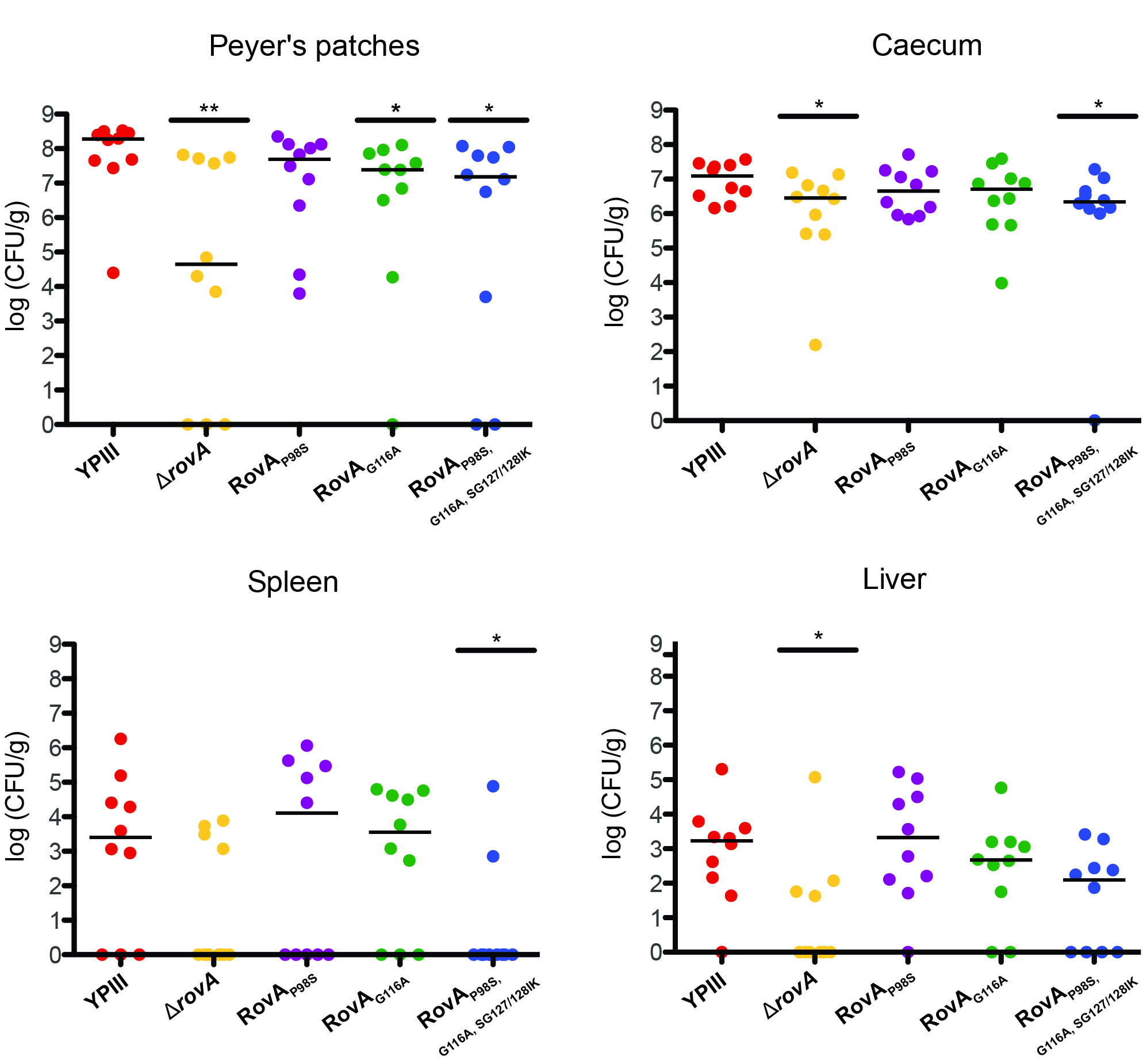

Supplement: S7 Fig — Oral infection of female Balb/c mice with 2 x 10^8^ bacteria producing wild-type RovA (YPIII), no RovA (ΔrovA) or more stable RovA variants (RovAP98S, RovAG116A, RovAP98S,G116A,SG127/128IK) led to reduced colonization of Peyer’s patches, caecum and spleen 3 days post infection (**, p < 0.01; *, p < 0.05; two-tailed Mann-Whitney test; n = 10 for each genotype and tissue). (JPG) [file ppat.1006091.s007.jpg]
