# Supplementary material for: A Precise Temperature-Responsive Bistable Switch Controlling Yersinia Virulence
Source: PLoS Pathog. 2016 Dec 22;12(12):e1006091. doi: 10.1371/journal.ppat.1006091 (PMC5179001; doi:10.1371/journal.ppat.1006091)
Supplement: S1 Table — This table lists and describes all plasmids and strain used in this study, including their sources and references. (DOCX) [file ppat.1006091.s008.docx]

**Table S1:** **Bacterial strains and plasmids**.

| **Strains, plasmids** | **Description** | **Source and reference** |
| --- | --- | --- |
| Bacterial strains |  |  |
| *E. coli* K-12 |  |  |
| DH10β | F^-^ *endA1 recA1 galE15 galK16* *nupG rpsL* ∆*lacX74* Φ80*lacZ* ∆M15 *araD139∆*(*ara, leu*)7697 *mcrA* ∆(*mrr-hsdRMS-mcrBC*)λ^-^ | [1] |
| BL21λDE3 | F^-^ *gal met r^-^m^-^ lon hsdS* λ_Lys_p*lac*UV5-T7 gene1 p*lacI*^q^ *lacI* | [2] |
| S17-1λpir | *recA1 thi pro hsdR^-^* RP4-2Tc::Mu Km::Tn7 λpir | [3] |
| *Y. pseudotuberculosis* |  |  |
| YPIII | pYV, wild-type | [4] |
| YP9  YP72 | YPIII, Δ*invA*  YPIII, Δ*rovM* | [5]  [6] |
| YP107 | YPIII, Δ*rovA* | [7] |
| YP269 | YP107, *rovA*^P98S^ | This study |
| YP270 | YP107, *rovA*^G116A^ | This study |
| YP287 | YP107, *rovA*^P98S, G116A, SG127/128IK^ | This study |
|  |  |  |
| Plasmids |  |  |
| pAKH120 | pACYC184, , P*_tet_*-*csrB,* Cm^R^ | [8] |
| pDM4 | R6K derivate, *sacB*, Cm^R^ | [9] |
| pFS5 | pKH70, P*rho-egfp_LVA_* | This study |
| pFS6 | pJet1.2, *rovA*, Amp^R^ | This study |
| pFS7 | pJet1.2, *rovA*_P98S_, Amp^R^ | This study |
| pFS8 | pDM4 *rovA*_P98S_, Cm^R^ | This study |
| pFS14 | pJet1.2, *rovA*_G116A_, Amp^R^ | This study |
| pFS16 | pDM4 *rovA*_G116A_, Cm^R^ | This study |
| pFS23 | pJet1.2, *rovA*_P98S, G116A_, Amp^R^ | This study |
| pFS24 | pJet1.2, *rovA*_P98S,G116A,SG127/128IK_, Amp^R^ | This study |
| pFS28 | pDM4 *rovA*_P98S,G116A,SG127/128IK_, Cm^R^ | This study |
| pFS42 | pFU76, P*_LtetO_-1*-*dsRed*, Kan^R^ | This study |
| pFS43 | pFS42, P*_LtetO_-1-mCherry*, Kan^R^ | This study |
| pFS48 | pFS43, p15a ori, Cm^R^ | This study |
| pFU33 | promoterless *luxCDABE*, p29807ori, Amp^R^ | [10] |
| pFU76 | promoterless *dsRed2*, R6K ori, Amp^R^ | [10] |
| pFU221 | promoterless *egfp_LVA_*, ColE1 ori, Amp^R^ | [10] |
| pFU228 | P*_gadh_*-*dsRed2*, ColE1 ori, Cm^R^ | [11] |
| pHT105 | pZS*24, P*_LtetO-1_*, Kan^R^ | [12] |
| pJet1.2 | cloning vector, Amp^R^ | Thermo Scientific |
| pKH31 | pHT105, P*_LtetO-1_*-*rovM*^+^, Kan^R^ | [12] |
| pKH70 | pKH87, ori29807, Amp^R^ | This study |
| pKH87 | pFU76, P*rovA*::*egfp*_LVA_, containing a *rovA* promoter fragment from -622 to +170, Amp^R^ | This study |
| pLW2 | pET28a, *rovA*-*his6*^+^, Kn^R^ | [13] |
| pTB23 | *mCherry*, attλ, ori R6K, Cm^R^ | [14] |
| pYPL | Kn^R^ pPROBE-*egfp*_LVA_ containing  *rovA* promoter fragment  from nt -622 to 170, Kan^R^ | [15] |
| pZE21 | P*_LtetO-1_*, Kan^R^ | [16] |

**References**:

1. Casadaban MJ, Cohen SN. Analysis of gene control signals by DNA fusion and cloning in *Escherichia coli*. J Mol Biol. 1980;138(2):179-207. PubMed PMID: 6997493.

2. Studier FW, Moffatt BA. Use of bacteriophage T7 RNA polymerase to direct selective high-level expression. J Mol Biol. 1986;189:113-30.

3. Simon R, Priefer U, Puehler A. A broad host range mobilization system for *in vivo* genetic engineering: transposon mutagenesis in gram negative bacteria. Biotechnology. 1983;1:784-91.

4. Bolin I, Norlander I, Wolf-Watz H. Temperature-inducible outer membrane protein of *Yersinia pseudotuberculosis* and *Yersinia enterocolitica* is associated with the virulence plasmid. Infect Immun. 1982;37:506-12.

5. Marra A, Isberg RR. Invasin-dependent and invasin-independent pathways for trans­location of *Yersinia pseudotuberculosis* across the Peyer's patch intestinal epithelium. Infect Immun. 1997;65(8):3412-21.

6. Heroven AK, Sest M, Pisano F, Scheb-Wetzel M, Steinmann R, Bohme K, et al. Crp induces switching of the CsrB and CsrC RNAs in *Yersinia pseudotuberculosis* and links nutritional status to virulence. Front Cell Infect Microbiol. 2012;2:158. Epub 2012/12/20. doi: 10.3389/fcimb.2012.00158. PubMed PMID: 23251905; PubMed Central PMCID: PMC3523269.

7. Quade N, Mendonca C, Herbst K, Heroven AK, Ritter C, Heinz DW, et al. Structural basis for intrinsic thermosensing by the master virulence regulator RovA of *Yersinia*. J Biol Chem. 2012;287:35796-803. doi: 10.1074/jbc.M112.379156. PubMed PMID: 22936808.

8. Heroven A, Bohme K, Rohde M, Dersch P. A Csr-type regulatory system, including small non-coding RNAs, regulates the global virulence regulator RovA of *Yersinia pseudo­tuberculosis* through RovM. Mol Microbiol. 2008;68(5):1179-95. Epub 2008/04/24. doi: MMI6218 [pii] 10.1111/j.1365-2958.2008.06218.x. PubMed PMID: 18430141.

9. Böhme K, Steinmann R, Kortmann J, Seekircher S, Heroven AK, Berger E, et al. Concerted actions of a thermo-labile regulator and a unique intergenic RNA thermosensor control *Yersinia* virulence. PLoS Pathog. 2012;8(2):e1002518. Epub 2012/02/24. doi: 10.1371/journal.ppat.1002518. PubMed PMID: 22359501; PubMed Central PMCID: PMC3280987.

10. Uliczka F, Pisano F, Kochut A, Opitz W, Herbst K, Stolz T, et al. Monitoring of gene expression in bacteria during infections using an adaptable set of bioluminescent, fluorescent and colorigenic fusion vectors. PLoS One. 2011;6(6):e20425. doi: 10.1371/journal.pone.0020425. PubMed PMID: 21673990; PubMed Central PMCID: PMC3108616.

11. Schweer J, Kulkarni D, Kochut A, Pezoldt J, Pisano F, Pils MC, et al. The cytotoxic necrotizing factor of *Yersinia pseudotuberculosis* (CNFY) enhances inflammation and Yop delivery during infection by activation of Rho GTPases. PLoS Pathog. 2013;9(11):e1003746. Epub 2013/11/19. doi: 10.1371/journal.ppat.1003746. PubMed PMID: 24244167; PubMed Central PMCID: PMC3820761.

12. Herbst K, Bujara M, Heroven AK, Opitz W, Weichert M, Zimmermann A, et al. Intrinsic thermal sensing controls proteolysis of *Yersinia* virulence regulator RovA. PLoS Pathog. 2009;5(5):e1000435. Epub 2009/05/27. doi: 10.1371/journal.ppat.1000435. PubMed PMID: 19468295; PubMed Central PMCID: PMC2676509.

13. Tran HJ, Heroven AK, Winkler L, Spreter T, Beatrix B, Dersch P. Analysis of RovA, a transcriptional regulator of *Yersinia pseudotuberculosis* virulence that acts through antirepression and direct transcriptional activation. J Biol Chem. 2005;280:42423-32.

14. Cox RS, 3rd, Dunlop MJ, Elowitz MB. A synthetic three-color scaffold for monitoring genetic regulation and noise. J Biol Eng. 2010;4:10. doi: 10.1186/1754-1611-4-10. PubMed PMID: 20646328; PubMed Central PMCID: PMCPMC2918530.

15. Lawrenz MB, Miller VL. Comparative analysis of the regulation of *rovA* from the pathogenic yersiniae. J Bacteriol. 2007;189(16):5963-75. PubMed PMID: 17573476.

16. Lutz R, Bujard H. Independent and tight regulation of transcriptional units in *Escherichia coli* via the LacR/O, the TetR/O and AraC/I1-I2 regulatory elements. Nucleic Acids Res. 1997;25(6):1203-10. PubMed PMID: 9092630.
