# Supplementary material for: A Precise Temperature-Responsive Bistable Switch Controlling Yersinia Virulence
Source: PLoS Pathog. 2016 Dec 22;12(12):e1006091. doi: 10.1371/journal.ppat.1006091 (PMC5179001; doi:10.1371/journal.ppat.1006091)
Supplement: S2 Table — This table lists all primers and their sequences used in this study. The corresponding restriction sites are underlined. Highlighted in bold are nucleotides exchanged by Quick-Change mutagenesis of rovA. (DOCX) [file ppat.1006091.s009.docx]

**Table S2:** **Oligonucleotides**.

| Primer | Sequence |
| --- | --- |
| 135 | GCGGCGGTCGACCAACGTAGTCGGTGCCATCGG |
| 151 | GCGGCGTCTAGATATATTATCTACATCCATCTGGC |
| 153 | GCCGCGTCTAGATGCCGCCTTCCTGCAACTCG |
| 158 | GCCGCGGTCGACTGCCGCCTTCCTGCAACTCG |
| 178 | GGGCCCGTCGACTCGTATTTATTGCTATTATCC |
| 296 | GCCGCGGGATCCATTAACCTAATACGAGTATCC |
| II379 | AACGGAACAGTCTTCA**TCG**ATAATCGAACAGGT |
| II380 | ACCTGTTCGATTA**TCG**ATGAAGACTGTTCCGTT |
| II525 | GCGCGCGCGGCCGCTTAAGCTACTAAAGCGTAGTTTTCGTCGTTTGCTGCTTTGTATAGTTCATCCATGCCATG |
| II624 | GCAAAGAAATACTT**GCG**GGGATTTCATCGG |
| II625 | CCGATGAAATCCC**CGC**AAGTATTTCTTTGC |
| II626 | gatgaaattGCAGTGTTA**ATAAAG**CTAATCGATAAGC |
| II627 | GCTTATCGATTAG**CTTTAT**TAACACTGCAATTTCATC |
| III784 | GCGGCGGCATGCGCAAGGCGTTCAGGGAGC |
| III947 | GCGGTCGACGGCGTGCTAACGACAATGAC |
| IV483 | GCGGGTCGACAAAGGAGGAGCAATTGGAATCG |
| IV490 | GCGGGACGTCGCTAACACAGCGGTGGCCTCAAG |
| IV491 | GCGGGTCGACGGGTATGTCTTGAACTATGGTCGTG |
| V519 | GGGCGCGAGCTCCAGGAGTCCAAGCGAGCTC |
| V520 | GGGCGCGACGTCGATCACTACCGGGCG |
| V521 | GGGCGCCCTAGGCGAATTGAGGGGTACTGG |
| V522 | GGGCGCGAGCTCCCTCGCTCACTGACTCGC |
| V842 | GCGGGTCGACAAAATAAGGAGGAAAAAAAAATGGTT |
| V843 | GCGGGCGGCCGCTTATTATTATTTGTACAGCTCATCCAT |
| V99 | GCGGCGTCTAGACCGATGGTCAATTAATGCGCG |

The corresponding restriction sites are underlined. Highlighted in bold are nucleotides exchanged by Quick-Change mutagenesis of *rovA*.
