# Supplementary material for: A Precise Temperature-Responsive Bistable Switch Controlling Yersinia Virulence
Source: PLoS Pathog. 2016 Dec 22;12(12):e1006091. doi: 10.1371/journal.ppat.1006091 (PMC5179001; doi:10.1371/journal.ppat.1006091)
Supplement: S3 Table — Calculated DNA binding constants for the activating and repressive RovA binding site at 25°C and 37°C are presented with open and fixed Hill coefficients. (PDF) [file ppat.1006091.s010.pdf]

Table S3: Temperature-dependent DNA binding constants of RovA.

Site	Temperature	Binding constant [nM]		Hill coefficient	RSS
activating	25°C	k_a	6.01	2	0.053
**activating**	**25°C**	$\boldsymbol{k_a}$	**6.09**	**3**	**0.030**
activating	37°C	k_a	10.69	2	0.093
**activating**	**25°C**	$\boldsymbol{k_a}$	**10.23**	**3**	**0.018**
**repressive**	**25°C**	$\boldsymbol{k_r}$	**12.22**	**2**	**0.048**
repressive	25°C	k_r	11.45	3	0.167
**repressive**	**37°C**	$\boldsymbol{k_r}$	**42.67**	**2**	**0.045**
repressive	37°C	k_r	40.69	3	0.017

Calculated DNA binding constants for the activating and repressive RovA binding site at 25°C and 37°C with open and fixed Hill coefficients.
