# Supplementary material for: A Precise Temperature-Responsive Bistable Switch Controlling Yersinia Virulence
Source: PLoS Pathog. 2016 Dec 22;12(12):e1006091. doi: 10.1371/journal.ppat.1006091 (PMC5179001; doi:10.1371/journal.ppat.1006091)
Supplement: S4 Table — All parameters used in the deterministic model (left column) and the stochastic model (right column) describing the shift experiment are listed. (PDF) [file ppat.1006091.s011.pdf]

Table S4: Model parameters.

name	value (det. model)	name	value (stoch. model)
		ζ	$1.45\ \mathrm{molecules/nM}$
$h_a(25°\mathrm{C})$	3	$\tilde{h}_a(25°\mathrm{C})$	(no change)
$h_a(37°\mathrm{C})$	3	$\tilde{h}_a(37°\mathrm{C})$	(no change)
$k_a(25°\mathrm{C})$	$6.09\ \mathrm{nM}$	$\tilde{k}_a(25°\mathrm{C})$	$\zeta\, k_a(25°\mathrm{C})$
$k_a(37°\mathrm{C})$	$10.23\ \mathrm{nM}$	$\tilde{k}_a(37°\mathrm{C})$	$\zeta\, k_a(37°\mathrm{C})$
$h_r(25°\mathrm{C})$	2	$\tilde{h}_r(25°\mathrm{C})$	(no change)
$h_r(37°\mathrm{C})$	2	$\tilde{h}_r(37°\mathrm{C})$	(no change)
$k_r(25°\mathrm{C})$	$12.22\ \mathrm{nM}$	$\tilde{k}_r(25°\mathrm{C})$	$\zeta\, k_r(25°\mathrm{C})$
$k_r(37°\mathrm{C})$	$42.67\ \mathrm{nM}$	$\tilde{k}_r(37°\mathrm{C})$	$\zeta\, k_r(37°\mathrm{C})$
α_0	$0.002\ \mathrm{nM/min}$	α_0	(no change)
α	$0.71\ \mathrm{nM/min}$	α	(no change)
		$\tilde{\alpha}$	$\zeta\alpha$
		$\tilde{\alpha}_0$	$\zeta\alpha_0$
δ_0	$5.2\ \cdot\ 10^{-6}/\mathrm{min}$		(no change)
δ_1	$0.2469/°\mathrm{C}$		(no change)

Parameters used in the deterministic model (left column) and the stochastic model (right column) describing the shift experiment.
