# Supplementary material for: A Precise Temperature-Responsive Bistable Switch Controlling Yersinia Virulence
Source: PLoS Pathog. 2016 Dec 22;12(12):e1006091. doi: 10.1371/journal.ppat.1006091 (PMC5179001; doi:10.1371/journal.ppat.1006091)
Supplement: S1 Text — This section includes the mathematical modelling approaches to simulate temperature-dependent bistability of the molecular thermometer RovA and the dynamics of the thermoresponsive bistable switch. It describes the determination of the parameters chosen for the simulation and includes a discussion of the models along with the experimental data of this study. (DOCX) [file ppat.1006091.s012.docx]

**Mathematical model approaches**

**Mathematical model approaches to simulate temperature-dependent bistability of the *Yersinia* virulence regulator RovA**

Our experimental approach demonstrated that synthesis of RovA – a proteinaceous thermometer that harbors an intrinsic thermosensor – is autoregulated by a positive and negative feedback loop and subjected to proteolysis (Fig. **1A**) [1, 2]. This control circuit results in a bistable behavior leading to a distinct distribution of RovA^+^ (ON) and RovA^-^ (OFF) bacterial cells within a certain temperature range (Fig. **1B-D**), whereby activation and deactivation of RovA synthesis follow very different time scales in response to thermal shifts (Fig. **2A-B**).

In the following analysis we use (i) a **deterministic model** to simulate the tem­poral change of RovA concentrations (d*r/*d*t*) in response to temperature by a bi­furcation and sensitivity analysis to identify the key features of the genetic switch and the bistable behavior of the regulatory system, and (ii) a **stochastic model** to describe the dynamics of the process in order to explain the different time scales observed for activation and deactivation of RovA synthesis and the heterogeneity of the bacterial population.

## **Part 1: Deterministic approach modeling the temperature-responsive beha­vior of RovA**

The applied deterministic modeling approach to describe the changes of RovA in response to temperature is based on one ordinary differential equation (ODE). Based on the experimental data, we assume that the temporal change of the production of RovA is dependent on itself, the temperature and a set of kinetic parameters and is given by

$\frac{dr}{dt}=\alpha0+\alpha\frac{r^{ha}}{{ka(T)}^{ha}+r^{ha}} \cdot\frac{{kr(T)}^{hr}}{{kr(T)}^{hr}+r^{hr}}- \delta\left( T \right) \cdot r$ (1)

In our model, $r$ denotes the concentration of RovA, and $T$ denotes the temperature. $\alpha$ represents the maximal increase of the production rate upon induction and $\alpha0$ the basal production rate [3]. A Hill function was used to describe the positive feedback loop with binding constant $ka$ and Hill coefficient $ha$, and the negative feedback was included into the model by an inhibitory Hill function with binding constant $kr$ and Hill coefficient $hr$. Cooperative DNA binding of the RovA dimer is described by the Hill coefficients $ha$ and $hr$. The degradation and dilution rate of RovA is given by $\delta$. Both, the binding constants and degradation rate of RovA are temperature-dependent [1, 2], and thus a function of temperature $T$ ($ka\left( T \right), kr(T)$ and $\delta(T)$).

## **Part 2: Stochastic approach to model the dynamics of the thermo-responsive bistable switching of RovA**

The following section describes a stochastic branching process corresponding to the deterministic model. The model allows to address the variability of active RovA molecules in a bacterial cell population, in particular in response to thermal shifts [4, 5]. This approach was chosen to capture the heterogeneity in the data, as the distribution of the RovA concentration per cell appears to be bimodal in certain situations (Fig. **1B-D**, Fig. **2A-B**). The deterministic model addresses a molecular concentration (nM), while the stochastic model relies on molecule numbers. The number of molecules per nM (de­fined as $\zeta$) was computed on the basis of the volume of a bacterial cell of appro­ximately to be

$$\zeta= 1.45 \frac{\mathrm{molecules}}{\mathrm{nM}}$$

$( 1 nM={\frac{{10}^{-9}N_{A}}{l} \mathrm{molecules}; N}_{A} = 6.022045\cdot{10}^{23}$ Avogadro-constant).

Let tilde denote the parameter values for $ka$, $kr$, $\alpha$, $\alpha_{0}$ relating to molecule numbers: $\tilde{k_{a}}=\zeta k_{a}$*, $\tilde{k_{r}}=\zeta k_{r}$, $\tilde{\alpha}=\zeta\alpha$,* $\tilde{\alpha}_{0}=\zeta\alpha_{0}.$ The parameters $h_{a}$, $h_{r}$ and $\delta\left( T \right)$ are not affected by this change of units. Let $X_{t}$ denote the number (random variable) of RovA molecules within the cell at time *t*. The production rate $\mathrm{prod}(X)$ of molecules in terms of molecules/time interval is given by

$$\mathrm{prod}\left( X \right)=\tilde{\alpha} 0+\tilde{\alpha} \frac{\tilde{r}^{ha}}{{\tilde{k}a\left( T \right)}^{ha}+\tilde{r}^{ha}} \cdot\frac{{\tilde{k}r(T)}^{hr}}{{\tilde{k}r(T)}^{hr}+\tilde{r}^{hr}}$$

and the degradation rate (molecules/time interval) by $\delta X=\delta\left( T \right)X$*.* In the following, we differentiate between the production rate $\mathrm{prod}\left( X \right)$ and the net production rate

$\mathrm{prod}(X)-\delta X$*.*

The stochastic branching model can be stated by

(2)

(3)

($\Delta t$ denotes a small time interval, and $O({\Delta t}^{2})$ denotes the Landau notation, $\lim_{x\to0}|O(x)|/x<\infty$).

The stochastic process can either be directly simulated e.g. by the Gillespie-algorithm [6], or alternatively, master equations can be used to approach the system [4]: Let $p_{i}\left( t \right)$ denote the probability to find a cell in a state with $i$ molecules at time $t$, $p_{i}\left( t \right)=P(X_{t}=i)$. Then, the master equation of the branching process is given by

$$\frac{d}{dt}p_{i}\left( t \right)=-\left[ \mathrm{prod}\left( i \right)+\delta i \right]p_{i}\left( t \right)+\mathrm{prod}\left( i-1 \right)p_{i-1}\left( t \right)+\delta\left( i+1 \right)p_{i+1}\left( t \right),$$

$$i\in{lN}_{0}$$

for which we formally defined $p_{-1}=0$. Notably, $\delta$ as well as $\mathrm{prod}\left( . \right)$ depend on the temperature $T$; if the temperature is changed during the experiment, the master equations are not autonomous. They form an infinite system of ordinary differential functions, which cannot be simulated directly. For the simulations, we restricted the number of RovA molecules to be smaller or equal to $\hat{N}$ in the system. There are several possible methods, e.g. to consider $X_{t}$ conditioned on staying $\in\{0,\ldots,\hat{N}\}$. In the present case, we set the production rate at $\hat{N}$ to zero, such that $X_{t}$ never exceeded this number, and found (e.g. with $i=1,\ldots\hat{N}-1$)

$\frac{d}{dt}p_{0}\left( t \right)=-\mathrm{prod}\left( 0 \right)p_{0}\left( t \right)+\delta p_{1}\left( t \right),$ (4)

$\frac{d}{dt}p_{i}\left( t \right)=-\left[ \mathrm{prod}\left( i \right)+\delta i \right]p_{i}\left( t \right)+\mathrm{prod}\left( i-1 \right)p_{i-1}\left( t \right)+\delta\left( i+1 \right)p_{i+1}\left( t \right),$ (5)

$\frac{d}{dt}p_{\hat{N}}\left( t \right)=-\delta\hat{N}p_{i}\left( t \right)+\mathrm{prod}\left( \hat{N}-1 \right)p_{\hat{N}-1}\left( t \right)$ (6)

For the simulations a cut off at $\hat{N}=80$ was chosen. We note that the net production rate that basically defines the average drift in the number of RovA molecules becomes negative for large values of $X$. The system is only able to enter this region by stochastic effects. In order to reach high numbers of RovA, random effects have to balance a deterministic, negative drift (in average more RovA molecules are degraded than produced), which is extremely unlikely, i.e. $p_{\hat{N}}\ll1$. Consequently, the present approximation does not introduce a serious error and is sufficient for a reasonable approximation. The mean value of RovA molecules can be computed by

Standard theory shows that the present stochastic process tends to the ODE under appropriate rescaling [7].

# **Part 3: Model parameters values and rates**

The following section describes the determination of the parameters chosen for the simulations in this study.

## *Temperature-dependent degradation rate of RovA*

Degradation rates of RovA were determined at 25°C, 28°C, 31°C, 34°C and 37°C. *Y. pseudotuberculosis* was grown in LB medium to the exponential phase and protein synthesis was stopped by addition of chloramphenicol. Samples were taken at *t* = 0, 30, 60 and 90 min, and processed for western blotting as described [1]. The RovA protein bands detected at the different temperatures ([1] and Fig. **S2A**) were quantified with the software ImageJ [8]. The degradation rate $\delta$ for each tempe­rature was determined according to the equation (Fig. **S2B**). Based on these data a non-linear regression with the function was performed. The resulting function for the temperature-dependent degra­dation rate

(8)

is shown in Fig. **S2C** demonstrating that the degradation rate of RovA increases strongly with temperature.

## *Temperature-dependent DNA binding constants of RovA*

Electrophoretic mobility gel shift assays (EMSA) were performed for the activating and the repressing binding site within the *rovA* regulatory region as described[1]. Titration of increasing RovA concentrations leads to a sigmoid curve of bound protein-DNA complex from which it is possible to derive the binding constants *k_d_* (half-saturation constants) (Fig. **S2D-H)**. In terms of the deterministic model developed above, the binding saturation $\nu$ (fraction of bound DNA) is given by the equation

$\nu=\frac{r^{h}}{r^{h}+k_{d}^{h}}$ . (9)

Remarkably, the saturation level of the repressive site was not reached even at high RovA concentrations at 37°C (Fig. **S2H**), indicating that RovA has a very low affinity to this binding site under these growth conditions.

Binding constants were determined for the activating and repressive binding sites $k_{a}$ and $k_{r}$. Non-linear regression was performed using equation (**9**) with integer Hill coefficients $h$. To measure the suitability of the fit to the data, the residual sum of squares (RSS) was calculated, which is the discrepancy of the data and the values predicted by an estimation model. The results are summarized in Table **S3**. Hill coefficients, $h_{a} = 3$ and $h_{r} = 2$, used in the simulations were chosen on the basis of a small RSS independent of the temperature, since the influence on the regression accuracy was negligible (Table **S3**).

To describe the temperature dependence of both binding constants, $k_{a}$ and $k_{r}$ were extrapolated using exponential functions

$k_{a}\left( T \right)= \lambda_{r} e^{\mu_{a}T}$ and $k_{r}\left( T \right)= \lambda_{a} e^{\mu_{r}T}$ (10)

with

$$\mu_{a}=\frac{\ln(k_{a}(T_{2})/k_{a}(T_{1}))}{T_{2}-T_{1}}=\frac{\ln(10.23/6.09)}{37^{\circ}C-25^{\circ}C}=0.04/^{\circ}C$$

$$\lambda_{a}=\frac{k_{a}(T_{1})}{e^{\mu_{a}T_{1}}}=\frac{6.09 nM}{e^{\mu_{a}25^{\circ}}}=2.07 \mathrm{nM}$$

$$\mu_{r}=\frac{\ln(k_{r}(T_{2})/k_{r}(T_{1}))}{T_{2}-T_{1}}=\frac{\ln(42.67/12.22)}{37^{\circ}C-25^{\circ}C}=0.1/^{\circ}C$$

$$\lambda_{r}=\frac{k_{r}(T_{1})}{e^{\mu_{r}T_{1}}}=\frac{12.22 nM}{e^{\mu_{r}25^{\circ}}}=0.9 \mathrm{nM}.$$

This non-linear approach was chosen to avoid negative values at lower tempera­tures, which would be the result of a linear fit (Fig. **S2I,J)**.

## *Estimation of RovA production rates*

In order to obtain the basal expression rate $\alpha_{0}$ and the maximal expression rate $\alpha$ of RovA, data of the temperature shift experiment (Fig. **2A,B**) were fitted by the sto­chastic model (equation (**7**)**,** Fig. **3A-B**, Fig. **S3A**) with the assumption that the reaction of the regulatory system to the thermal downshift is delayed by 1 h. RovA abundance and *rovA* promoter activity were determined via western blotting and the P*_rovA_*-*egfp_LVA_* reporter. RovA activity was determined at 37°C for 8 h and after a temperature shift to 25°C for 18 h (Fig. **2A-B**).

Expression rates revealed $\alpha=0.711 nM/min$ and $\alpha_{0}=0.002 nM/min$. Figure **S3A** illustrates that a distinct minimum of the deviance was obtained for these production rates. Further, RovA molecule numbers per cell at 25°C were determined via western blotting of YPIII P*_rovA_*-*egfp_LVA_* and defined amounts of recombinant His-tagged RovA (RovA-His_6_) (Fig. **S3B**). Per cell an average of 400 RovA molecules were measured, which corresponds to approximately 275 nM RovA per cell. The parameters $\alpha$ and $\alpha_{0}$ predict a lower concentration of around 35 nM of free RovA (≈25 RovA dimers based on the previous calculation of RovA molecules in the cell per nM defined as $\zeta$). This is expected, since not all RovA molecules within the bacterial cell are available for autoregulation. Only a certain part of the RovA molecules are in the active form and a certain number of RovA dimers are also likely to be bound at different locations on the bacterial chromosome. Furthermore, the entire bacterial population is still in the ON state at 30°C (Fig. **1D**), while RovA amounts are considerably decreased compared to 25°C (40-50%), indicating that less than 275 nM of RovA is sufficient to trigger RovA autoinduction in the entire population.

To further validate the stochastic model, we compared the distribution of RovA according to the stochastic model based on the flow cytometry data (i.e. distribution of RovA ON and OFF cells) at measured time points of the start of the temperature shift ex­periment (= 0 h). We found a remarkable similarity between the model and the data (Fig. **S3C**). All calculated parameters are shown in Table **S4**.

In addition, a stimulus response diagram was generated illustrating the steady-state concentration of RovA at 25°C-60°C for the calculated maximal and basal expression rates (Fig. **3C**). A bistable response behavior of the *rovA* promoter was detected within a temperature range from 28°-36°C which reflects the experimental data (Fig. **1**). Furthermore, observed experimental differences for activation and deactivation of *rovA* expression (hysteresis) after a thermal downshift or upshift (Fig. **2A-B**) could be reproduced with the models (Fig. **3A-B**).

# **Part 4: Analysis of the temperature-responsive bistability**

## *Analysis of bistability*

A numerical approach was used to analyze bistable expression of *rovA* depending on the temperature $T$, the degradation rate $\delta$ and the production rates $\alpha$ and $\alpha_{0}$. For this purpose the degree of bistability was defined as the absolute difference between the minimal and maximal RovA concentrations in all stationary states present for a given temperature (note that this difference becomes zero outside the bistable region) and plotted as colored levelplots. The resulting state diagrams visualize the influence of $\delta$, $\alpha$ and $\alpha_{0}$ on bistability (Fig. **3D**).

As the degradation rate $\delta$ is a function of temperature (Fig. **S2A-C**), the degra­dation rates cross the bistability area and enter the monostable states at the tem­perature extrema. Extremely high and low degradation rates would abolish bistable behavior (Fig. **3D**), illustrating that temperature de­pendence of the degra­dation rate is crucial for the bistable behavior of RovA.

With an increasing induced production rate $\alpha$ the degree of bistability increases. Moreover, the temperature range in which bistability is observed is only slighty shifted (Fig. **3D**), indicating that bistability of RovA is robust to small changes of $\alpha$. Moreover, the basal production rate $\alpha_{0}$ is critical for RovA bistability. In case $\alpha_{0}$ is too high, the system remains in a monostable state (Fig. **3**), whereas if $\alpha_{0}=0$, the system is no longer able to switch.

## *Influence of the inhibitory RovA binding site*

In order to analyze the influence of the inhibitory RovA binding site on bistable ex­pression of *rovA*, the part including the negative feed back control was removed from equation 1, mimicking a potential mutant harboring a deletion of the inhibitory binding site, which results in:

$\frac{dr}{dt}= \alpha_{0}+\alpha\frac{r^{h_{a}}}{{k_{a}(T)}^{h_{a}}+r^{h_{a}}}- \delta\left( T \right)\cdot r.$ (11)

Surprisingly, the range of bistability of this *in silico* mutant was not altered when com­pared with the wild-type system (Fig. **S4A-B**). However, the fine-tuning of RovA levels at lower temperatures and the robustness of the stable states are dramatically affected. The comparison of the levelplots revealed that only the level of bistability is altered while the parameter space of bistability remains nearly identical.

## *Transient state and time scales*

Our analysis further showed that presence of the inhibitory binding site of RovA de­creased the response time to a temperature change, in particular to a thermal upshift (Fig. **S4C-D**). Moreover, the response time after a shift from 37°C to moderate tem­perature within the bistability region becomes arbitrarily long. This might improve the responsiveness of the system upon a sudden thermal upshift sensed during host entry and increases its robustness at host temperatures against temporal thermal fluctuations.

## *Mechanism responsible for the long lag phase following a thermal downshift*

We hypothesize that the extended lag phase of *rovA* activation after a thermal down­shift is based on the fact that only very few active RovA molecules are present in the bacteria at 37°C which are not able to activate the positive feedback loop. We propose that in this case the RovA population follows initially a stochastic birth-death process with a constant birth- and death rate where the positive feedback loop is not active. Once (by chance) a certain threshold is reached the positive feedback loop takes over and the cells rapidly go to the ON state (in accordance with the bimodal distribution shown by the data depicted in Fig. **2A-B, 3A-B**).

In order to build the model, we considered a random walk on $\{0,\ldots,N\}$ RovA mole­cules. Let $X$*_t_* be the number of RovA molecules at time *t*. The net production rate $\tilde{\alpha}_{0}$ was considered to be constant independent of the number of RovA molecules present in the cell. The degradation rate was given by $\delta X_{t}$. If our random walk hits $X_{t}=N$, we assumed that the positive feedback dominates, and the cell becomes activated. Once the cells switched to the ON state, they stay activated (in this simple model and on the time scale we focused on in the present model). In summary, we were faced with a birth-death process for RovA molecules that has an absorbing state $N$ (the Markov chain cannot leave this state once it jumped into it).

Let $p_{i}\left( t \right)=P(X_{t}=i)$ be the probability to find the bacterial cell with $i$ RovA mole­cules,$i=0,\ldots,N-1$. Hence, the probability to find the cell to be activated (in the RovA ON state $X_{t}=N$) is given by

$$1-\sum_{i=0}^{N-1} p_{i\left( t \right).}$$

The differential equations for $p_{i(t)}$ read (for $i \epsilon\{1,\ldots,N-2\}$)

${\frac{d}{dt} p}_{0} (t)=- \tilde{\alpha}_{0} p_{0} (t)+ \delta p_{1} (t)$ (12)

(13)

(14)

We assumed that we start with zero RovA molecules at the end of the thermal upshift (time is $8 h$, $p_{0}\left( 8 h \right)=1, p_{i} \left( 8 h \right)=0$ for $i>0$). A simulation for $N=6$ is in full agree­ment with our experimental RovA data (Fig. **S3D-E)**. The probability to find a certain fraction of bacteria in the RovA ON state (in relative units, between $0,\ldots,1$) perfectly correlates with the increase of RovA ON cells determined by flow cytometry and western blotting (Fig. **2A-B, 3A-B**). This confirms our initial hypothesis and explains why *rovA* activation is significantly delayed in the majority of bacterial cells within a population after a thermal downshift.

**References**

1. Herbst K, Bujara M, Heroven AK, Opitz W, Weichert M, Zimmermann A, et al. Intrinsic thermal sensing controls proteolysis of *Yersinia* virulence regulator RovA. PLoS Pathog. 2009;5(5):e1000435. Epub 2009/05/27. doi: 10.1371/journal.ppat.1000435. PubMed PMID: 19468295; PubMed Central PMCID: PMC2676509.

2. Quade N, Mendonca C, Herbst K, Heroven AK, Ritter C, Heinz DW, et al. Structural basis for intrinsic thermosensing by the master virulence regulator RovA of *Yersinia*. J Biol Chem 2012;287:35796-803. doi: 10.1074/jbc.M112.379156. PubMed PMID: 22936808.

3. Yagil G, Yagil E. On the relation between effector concentration and the rate of induced enzyme synthesis. Biophys J. 1971;11:11-27.

4. Jagers P. Branching Processes with Biological Applications. : John Wiley; 1975.

5. Athreya K, Ney P. Branching Processes Springer; 1972.

6. Gillespie TD. A general method for numerically stimulating the stochastic time evalution of coupled chemical reactions. J Comput Phys. 1976;22:403-34.

7. Kurtz T. Relationship between stochastic and deterministic population models. Lecture Notes in Biomathematics. 1980:449 67.

8. Schneider CA, Rasband WS, Eliceiri KW. NIH Image to ImageJ: 25 years of image analysis. Nat Methods. 2012;9(7):671-5. PubMed PMID: 22930834.
